# Cyanide-bridged iron complexes as biomimetics of tri-iron arrangements in maturases of the H cluster of the di-iron hydrogenase†
†Electronic supplementary information (ESI) available: Experimental details, additional spectroscopic, electrochemical and computational details, and X-ray crystallographic data (CIF) from the structures of complexes **A–D**. CCDC 1447441–1447444. For ESI and crystallographic data in CIF or other electronic format see DOI: 10.1039/c6sc00213g


**DOI:** 10.1039/c6sc00213g

**Published:** 2016-02-29

**Authors:** Allen M. Lunsford, Christopher C. Beto, Shengda Ding, Özlen F. Erdem, Ning Wang, Nattamai Bhuvanesh, Michael B. Hall, Marcetta Y. Darensbourg

**Affiliations:** a Department of Chemistry , Texas A & M University , College Station , TX 77843 , USA . Email: marcetta@chem.tamu.edu; b Department of Physics , Middle East Technical University , 06800 Ankara , Turkey

## Abstract

Developing from certain catalytic processes required for ancient life forms, the H_2_ processing enzymes [NiFe]- and [FeFe]-hydrogenase (H_2_ase) have active sites that are organometallic in composition, possessing carbon monoxide and cyanide as ligands. Simple synthetic analogues of the 2Fe portion of the active site of [FeFe]-H_2_ase have been shown to dock into the empty carrier (maturation) protein, apo-Hyd-F, *via* the bridging ability of a terminal cyanide ligand from a low valent Fe^I^Fe^I^ unit to the iron of a 4Fe4S cluster of Hyd-F, with spectral evidence indicating CN isomerization during the coupling process (Berggren, *et al.*, *Nature*, 2013, **499**, 66–70). To probe the requirements for such cyanide couplings, we have prepared and characterized four cyanide-bridged analogues of 3-Fe systems with features related to the organoiron moiety within the loaded HydF protein. As in classical organometallic chemistry, the orientation of the CN bridge in the biomimetics is determined by the precursor reagents; no cyanide flipping or linkage isomerization was observed. Density functional theory computations evaluated the energetics of cyanide isomerization in such [FeFe]–CN–Fe ⇌ [FeFe]–NC–Fe units, and found excessively high barriers account for the failure to observe the alternative isomers. These results highlight roles for cyanide as an unusual ligand in biology that may stabilize low spin iron in [FeFe]-hydrogenase, and can act as a bridge connecting multi-iron units during bioassembly of the active site.

## Abstract 2

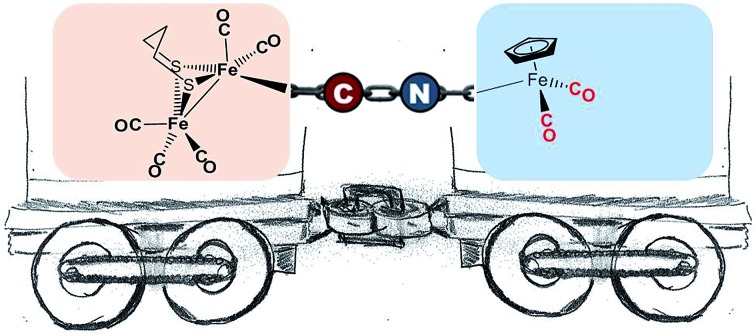
Concepts from organometallic chemistry are used to define possibilities of cyanide as a docking unit for bioassembly processes.

## Introduction

The discovery of cyano-iron carbonyls in the hydrogen processing enzymes [NiFe]- and [FeFe]-hydrogenase (H_2_ase) has inspired synthetic efforts by organometallic chemists to reproduce the active sites of these extraordinarily efficient H_2_-producing or H_2_-oxidation enzymes.^1^ The recent demonstration of the possibility of loading apo-HydA (the [FeFe]-H_2_ase enzyme *sans* the 2Fe subsite) and apo-HydF (a precursor protein in the maturation process) with synthetic analogues of the active site are convincing that small molecular models are indeed representative of the essential catalyst.^2^–^5^ Such “simple methodology of controlled metalloenzyme activation” as described by Berggren, *et al.*^2^ and Esselborn, *et al.*,^5^ provided unambiguous evidence that the bridgehead atom X of the S to S linker, μ-SCH_2_XCH_2_S, in the 2Fe-subsite is NH. The need for such a pendant base adjacent to an open site on Fe was already proposed from a theory-generated mechanism of heterolytic hydrogen production or cleavage, and corroborated by spectroscopy.^6^–^10^

**Fig. 1:**
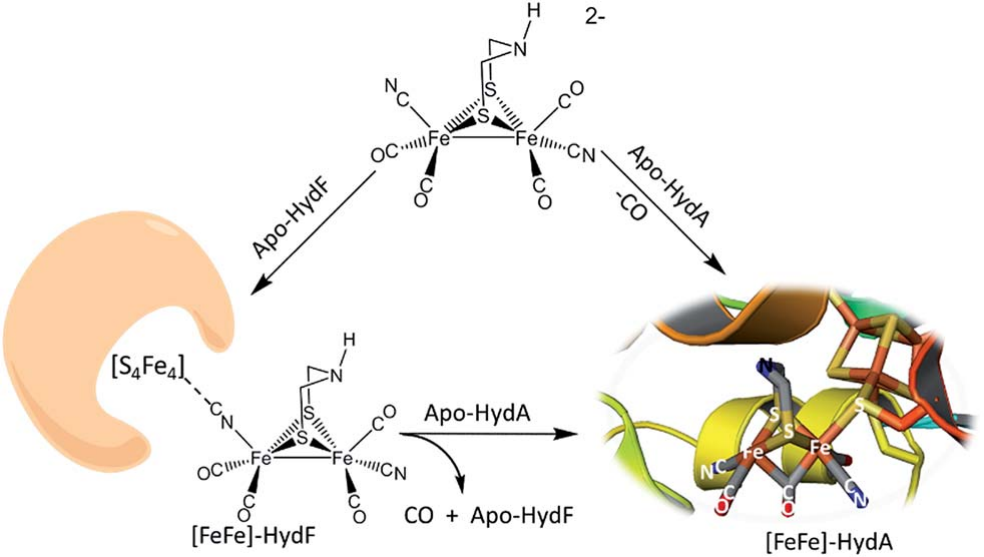
Hydrogenase precursor protein, HydF, loaded with synthetic analogues of the active site and hydrogenase enzyme, HydA.^2^,^3^

The interrogation using pulsed EPR spectroscopy of the Hyd-F maturase protein containing the 2Fe subsite derived from the synthetic analogue yielded an unexpected conclusion.^2^ As shown in Fig. 1, a linear cyanide bridges the [4Fe–4S] cluster to the 2Fe subsite; HYSCORE data found the unpaired electron of the [4Fe–4S] cluster to be strongly coupled to the C-13 nucleus of cyanide derived from the 2Fe unit prepared with labeled ^13^CN^–^. The molecular interpretation of this observation is that the cyanide that serves to couple the 2Fe unit to the HydF carrier protein has flipped from its origin, placing the cyanide nitrogen next to the 2Fe site. As the 2Fe unit is transferred to the apo-HydA another flip occurs, returning the CN^–^ to the C-bound terminal position. While examples of μ-CN^–^ linkage isomers between two transition metals are found in inorganic and organometallic chemistry, such maneuvers as CN linkage isomerism, *i.e.* CN flipping, subsequent to adduct formation are not common.^11^,^12^ Hence we have endeavoured to prepare cyanide-bridged constructs of 3-Fe systems with features related to the organoiron moiety within the loaded HydF protein. Further significance of such synthetic studies relates to the biosynthesis of the Fe(CO)_2_CN moiety itself, as fundamental background for cyanide interactions in iron-rich proteins.

## Results and discussion

### Synthesis and characterization

**Chart 1:**
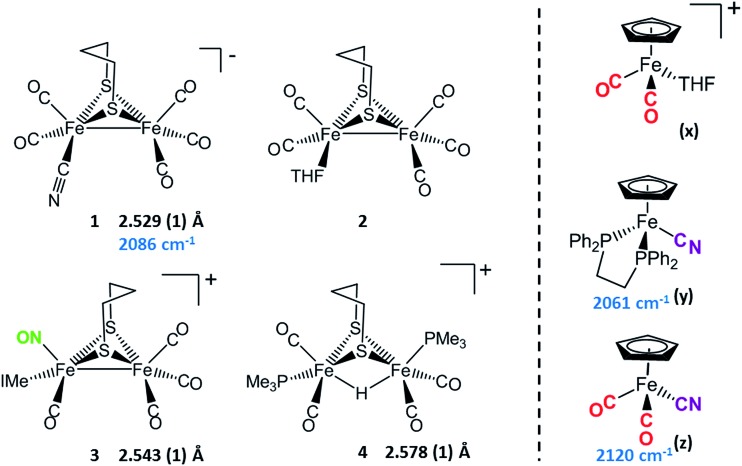
[FeFe] and [Fe] precursors to μ-CN Fe_3_ complexes; Fe–Fe distances (Å) and *ν*(CN) values, cm^–1^ listed where appropriate. IMe = 1,3-dimethylimidazole-2-ylidene.

**Fig. 2:**
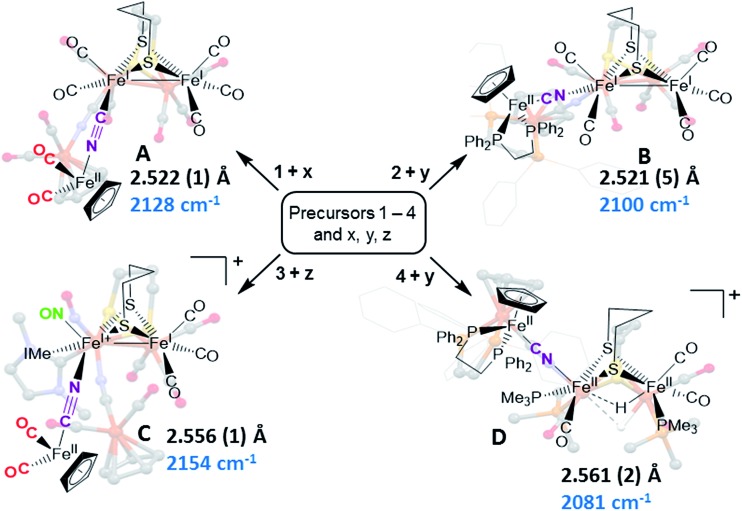
Combinations of [FeFe] and [Fe] precursors (from Chart 1) and products. Structures from XRD analysis are shown in relief. Full reports and TEPs of the four structures are given in the ESI.† Fe–Fe distances (Å) and *ν*(CN) values, cm^–1^, are listed with each structure.

Synthetic efforts were guided by reports from Zhu and Vahrenkamp for the synthesis of several organometallic complexes of the type M–CN–M′/M–NC–M′ using a labile ligand approach and judicious balance of the electropositive characters of M and M′.^13^ The precursors chosen for the di-iron complexes, complexes **1–4** in Chart 1, are noted to replicate certain features of the [FeFe]-H_2_ase enzyme active site; the cyclopentadienyl iron derivatives, **x**, **y**, and **z**, are well known Fe(ii) complexes, meant to mimic a ferrous iron in the 4Fe–4S cluster of HydF. Using these precursors, the four cyanide-bridged, 3-Fe complexes displayed in Fig. 2 were prepared, isolated in crystalline form and characterized using X-ray diffraction. Full structure reports are deposited in the ESI,† and details of isolation and purification are outlined in the experimental section. Note that the Fe–Fe distances in the di-iron moiety are similar in all, however the extent to which there is an Fe–Fe bond undoubtedly differs. For example, complex **D** is not expected to have the possibility of a formal bond due to the existence of the (semi-)bridging hydride.

Complex **A** was prepared from an eminent precursor for scores of [FeFe]-H_2_ase mimetics, (μ-pdt)[Fe(CO)_3_]_2_, here with one CO ligand substituted by CN^–^, namely complex **1**.^14^ When **1** is exposed to the labile ligand-containing complex **x**, (η^5^-C_5_H_5_)Fe(CO)_2_(THF)^+^, derived from halide abstraction of (η^5^-C_5_H_5_)Fe(CO)_2_Br in THF solvent, conversion to the cyanide-bridged product **A** was completed within minutes. The isolated neutral complex **A** is bench-top stable, even allowing column chromatography on silica without protection from air.

From the purified product, X-ray quality crystals were obtained and the corresponding diffraction data were refined as noted. With the cyanide C-bound to the di-iron precursor and N-bound to the mono-Fe^II^, an *R*_f_ of 3.17 was obtained. The opposite assignment led to elongated N ellipsoids and an *R*_f_ of 3.43. Such assignments of linkage isomers based on XRD refinements of both possibilities, M–CN–M′ or M–NC–M′ arrangements, are typical for the 22 organometallic complexes in the Vahrenkamp study, and commonly used in other cases.^11^–^13^ This approach is the basis for all CN linker assignments in our study as well, see Fig. S28–S31.†

As noted in Chart 1 and Fig. 2, there is a *ca.* 40 cm^–1^ shift to higher values of the *ν*(CN) in the product **A** in contrast to the precursor **1**, ascribed to the kinematic effect operative in bridging cyanides.^15^,^16^ There is also a gain of intensity in the *ν*(CN) over the precursor, consistent with the increased delocalization of π electron density in the Fe^I^–CN–Fe^II^ product. Minor positive shifts in the *ν*(CO) values of the carbonyls on the di-iron moiety are indicative of a drain of electron density *via* the interaction of the cyanide with the Fe(ii); consistently, the *ν*(CO) values of the (η^5^-C_5_H_5_)Fe(CO)_2_ appendage are shifted negatively. The diatomic ligand spectral changes resulting from the Lewis acid/base interaction of [Fe^I^Fe^I^]CN^–^, and the enhanced stability of **A** over **1**, are consistent with the properties of cyanide adducts of (μ-pdt)[Fe(CO)_2_CN]_2_^2–^ with BAr^F^_3_.^17^–^19^

Attempts to prepare the linkage isomer of **A** by addition of the (η^5^-C_5_H_5_)Fe(CO)_2_CN complex (**z**) to the labile solvent bound di-iron derivative **2** were unsuccessful. However, with increased basicity of the cyanide on precursor **y**, the (η^5^-C_5_H_5_)Fe(dppe)CN complex reacted with **2** generating **B** whose characterization using spectroscopy and XRD indicated the Fe^I^Fe^I^–NC–Fe^II^ arrangement shown in Fig. 2. Specifically the IR spectrum in the diatomic region found the *ν*(CN) value again shifted positively by 40 cm^–1^ compared to **y**; and the pattern complexity and slight negative shifts of the *ν*(CO) indicated the effect of the electron donating cyanometallate ligand on the di-iron moiety. In contrast to **A**, complex **B** is much less stable to air and moisture.

An alternative strategy to obtain the Fe^II^–CN–[FeFe] species is to enhance the electrophilicity of the di-iron unit. This we have done by two methods: complex **C** exploited the electrophilic properties of an unusual di-iron complex that has carbonyls on a single iron substituted by an N-heterocyclic carbene (NHC) and NO^+^.^20^ This asymmetric substitution pattern on precursor **3** has the effect of producing a “hot iron”, on which the electrophilicity of the nitrosonium ligand overwhelms the good electron-donating character of the NHC ligand. Subsequent addition of ligands of disparate donor properties (from ^13^CO to metallodithiolates of the MN_2_S_2_ class) results in replacing the remaining CO on that iron, which is of substantial Fe^II^ character.^20^ Thus, the mono-iron precursor (**z**) readily reacts with **3** to produce complex **C**.

The fourth member of the tri-iron series was generated by photolysis of **4**, (μ-pdt)(μ-H)[Fe(CO)_2_PMe_3_]_2_^+^ as its PF_6_^–^ salt, in the presence of **y**.^21^ As the oxidation state of the irons in the di-iron precursor **4** is formally +2, the precursor **z**, even without the dppe donor ligand, would likely have given a similar success. However, the photolysis route to CO loss in **4** also degrades CO-containing **z**, hence the photolysis-tolerant **y** was the better choice for the mono-iron precursor. Color changes (red-orange to dark green-brown) indicated reaction progress of **4** + **y**, and the *ν*(CN) value of **y**, 2061 cm^–1^, shifts to 2081 cm^–1^ in complex **D** (note that solution phase IR data comparing the diatomic ligand region spectral patterns and positions are compiled in Fig. S12 and Table S2† for all Fe_3_ complexes).

As indicated in Fig. 2, the μ-H of **D** is judged to be asymmetric (from the electron density maximum determined using crystallography, *vide infra*) and more tightly bound to the less substituted Fe^II^. Consistently, the hydride region of the ^1^H NMR spectrum shows a doublet of doublets due to coupling with the non-equivalent P-31 nuclei, Fig. S23.†

### Electrochemistry

**Fig. 3:**
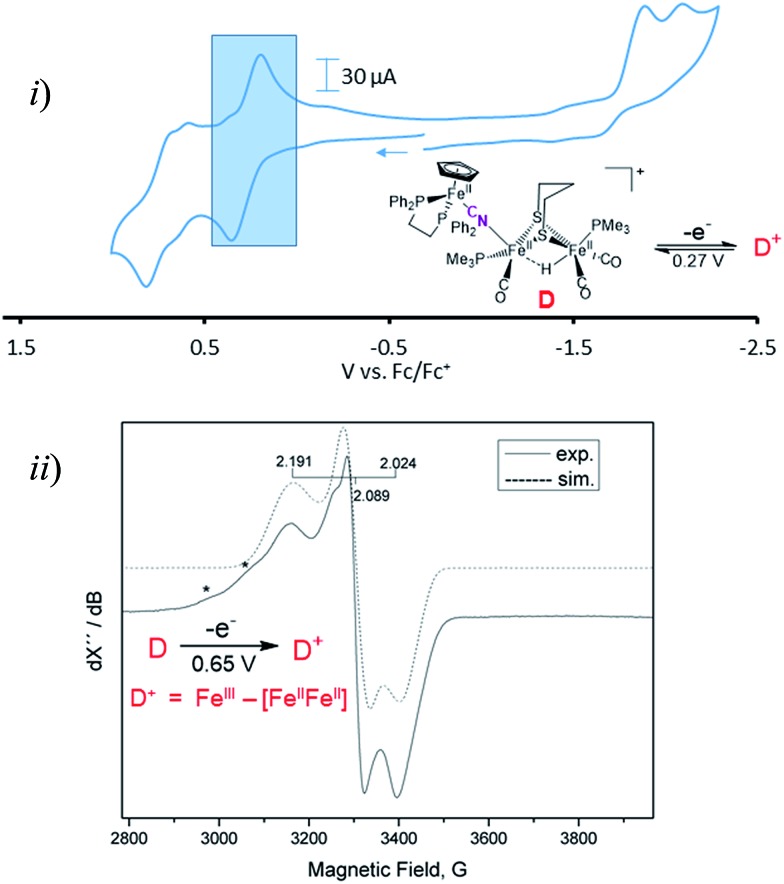
(i) Cyclic voltammogram of compound **D** in DCM at 200 mV s^–1^ referenced to Fc^0^/Fc^+^ = 0 V. The shaded region represents the oxidation event of the CpFe(dppe) unit at *E*_1/2_ = 0.27 V. (ii) Coulometric oxidation at 0.65 V generates a stable radical species with the representative X-band EPR spectrum shown. (*) denotes possible decomposition products.

Cyclic voltammograms for complexes **A–D** are found in Fig. S1–S4† and a representative scan of complex **D** is given in Fig. 3. Complex **A** in CH_3_CN exhibits three irreversible reduction events and two irreversible oxidation events within the range of +1.2 to –2.5 V, see Fig. S1 for full, and S8† for partial scans. Another oxidation at 0.1 V results from degradation prior to reduction, even at the mildest –1.35 V potential. Assignment of the redox events relies on earlier definitive electrochemical studies of CpFe(CO)_2_X species.^22^ Upon reduction, X^–^ is typically released and the resultant CpFe(CO)_2_˙ radical couples, yielding the [CpFe(CO)_2_]_2_ dimer, whose one electron reduction is at –2.03 V. Whether the most negative response at –2.23 V is from the adduct **A** or from the precursor to **A**, the monocyanide (**x**), is uncertain. The CVs of adducts **B** and **C** can similarly be described, however assignments are uncertain and redox-induced degradation is likely. Adduct **D** displays a reversible oxidation at 0.27 V that is more distinct when initially scanned in the positive direction and is assigned to the iron of the CpFe unit; the two irreversible reductive events are assigned to the di-iron unit.

Adduct **D** is the most stable of the four bridging cyanide complexes and its reversible oxidation suggested the possibility of generating an intact tri-iron radical observable using EPR spectroscopy. Coulometric oxidation of **D** at 0.65 V yielded an EPR-active species presumed to be **D^+^**. Its X-band EPR spectrum at 10 K displayed as a rhombic *g*-tensor, consistent with a low spin Fe^III^ center, with *g* (1, 2, 3) = (2.191, 2.089, 2.024). Additional minor signals at *g* = 2.254 and 2.312, are presumed to be decomposition products in the sample which are marked by asterisks in Fig. 3. Computations (DFT) found the majority of the unpaired spin to be on the iron of the mono-iron moiety, the CpFe(dppe) unit; anisotropic hyperfine coupling constants of *A*_iso_ = –98.65 and *A*_iso_ = –97.11 MHz for the two adjacent phosphorus atoms, Table S3,† were similar to the qualitative spectral simulation (with Easyspin^23^) of *A*_iso_ (^31^P)_1_ = –85 and *A*_iso_ (^31^P)_2_ = –77 MHz. The spin density contour plot from DFT calculations showed 1.163 e^–^ on the mono-iron of Fe^III^ character, while minor excess spins are on the two other irons, bridging cyanide and cyclopentadienyl unit, Fig. S25.† A hyperfine coupling pattern due to the ^14^N of the cyanide could not be observed in the EPR spectrum, indicating a small coupling constant. From ^14^N HYSCORE data obtained at 343.8 mT (corresponding to *g*_2_ = 2.089), this weak coupling to the nitrogen was found to be *A*_iso_ = 3 MHz, Fig. S26.† DFT calculations showed that if the nitrogen atom of the cyanide is two atoms away from the spin center, as expected for **D** and **D^+^**, the calculated isotropic coupling constant is 1.4 MHz, Table S3.† If the cyanide nitrogen was bound directly to the spin center the isotropic coupling of the nitrogen atom would increase to 9.5 MHz. This evidence suggests that the orientation of the cyanide in **D^+^** is the same as shown in Fig. 2 for its neutral **D** precursor.

### Computational protocols

Density functional theory computations were applied to evaluate the energetics of the possible linkage isomerization during the formation of M–CN–M′ bridges from precursors M–CN and M′. Crystal structures of complexes **A–D** were imported as the initial geometries of the experimentally observed isomers. Computational IR frequencies matched with experimental values supporting the validity of the computational methodology, see Table S2.† The initial geometries of their cyanide-flipped isomers were created by computationally exchanging the positions of C and N in the crystallographic structures. Geometric optimizations were done with Gaussian 09, as well as thermal and solvation corrections. Transition states were initially generated through relaxed scans with educated guesses and successively optimized. The Intrinsic Reaction Coordinate (IRC) calculations were applied to certain transition states, to trace the reaction paths following the imaginary vibrational frequency, until a local minimum on the potential energy surface was achieved. A detailed methodology description and the coordinates of the optimized structures are provided in the experimental section; Table S1† contains selected calculated metric data that compares to experimental values with approximately 1–2% error, lending confidence to the validity of the computations.

Despite the fact that the orientation of cyanide in these synthesized complexes is predetermined by the precursors, the calculations showed that it is always energetically advantageous for the carbon to coordinate to the mono-iron moiety in the four complexes. The Gibbs free energy differences between the two isomers of complexes **A–D** were determined to be 1.0, 2.0, 3.2 and 4.4 kcal mol^–1^, respectively.

**Fig. 4:**
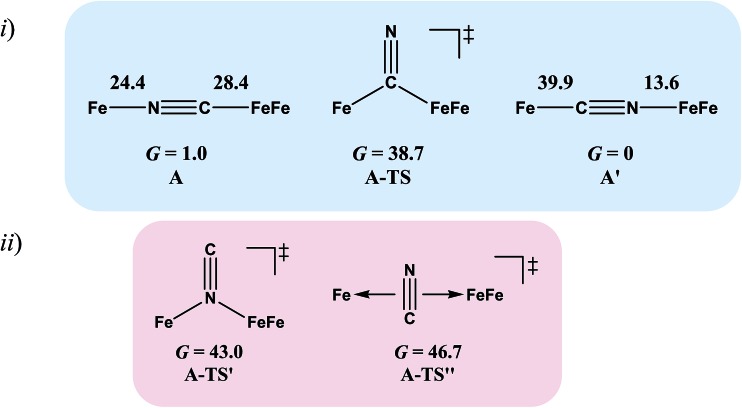
(i) Cyanide-flipped isomers of complex **A** and a possible transition state connecting them. Gibbs free energies Δ*G* of bond rupture of these species are given in kcal mol^–1^ with thermal and solvation corrections (solvent: acetonitrile). (ii) Other transition states that were optimized.

Complex **A**, the simplest structure, was investigated as a representative example to search for a cyanide-flipping mechanism. The C–Fe and N–Fe bond energies of the linkage isomers were calculated (Fig. 4i), finding that the carbon, rather than the nitrogen end of CN forms the stronger bond to either Fe^II^ or Fe^I^Fe^I^, see **A′** and **A** of Fig. 4i. The imaginary vibrational frequency of **A-TS**, located as the likely transition state connecting the isomers (see Fig. 4i), is associated with a wagging motion that initiates the asymmetric concomitant slide from one CN end to the other. The motion is indicative of an intramolecular transfer mechanism rather than a dissociation-association mechanism. The Gibbs free energy of **A-TS** was calculated to be 38.7 kcal mol^–1^ above the more stable isomer **A′**, a barrier unlikely to be overcome at room temperature, and thus consistent with the experimental observation of only one isomer.

While many features of the enzyme active site are mimicked in our model complexes, an obvious difference is the lack of an aza-dithiolate linker connecting the two sulfur atoms. Erdem *et al.* have shown that when the central atom of this linker is NH, it may be electronically influenced by the ligands attached to the iron.^24^ Presumably, the reverse would be true, *i.e.*, changing the atom from NH to CH_2_ may be expected to alter the electronics of the Fe–CN unit to some extent, and consequently the energy of cyanide isomerization. However, the replacement of the dithiolate linker in **A**, **A′** and **A-TS** by azadithiolate (adt, –SCH_2_NHCH_2_S–), *i.e.*, having the N bridgehead as in [FeFe]-hydrogenase, does not significantly change the DFT computed energetics. The **adt-A′** with an Fe–CN–FeFe sequence is still more stable, by 1.2 kcal mol^–1^, than **adt-A** with an Fe–NC–FeFe sequence. The corresponding transition state **adt-A-TS**, is 38.6 kcal mol^–1^ less stable than **adt-A′**, similar to the 38.7 kcal mol^–1^ for the pdt analogue.

**Fig. 5:**
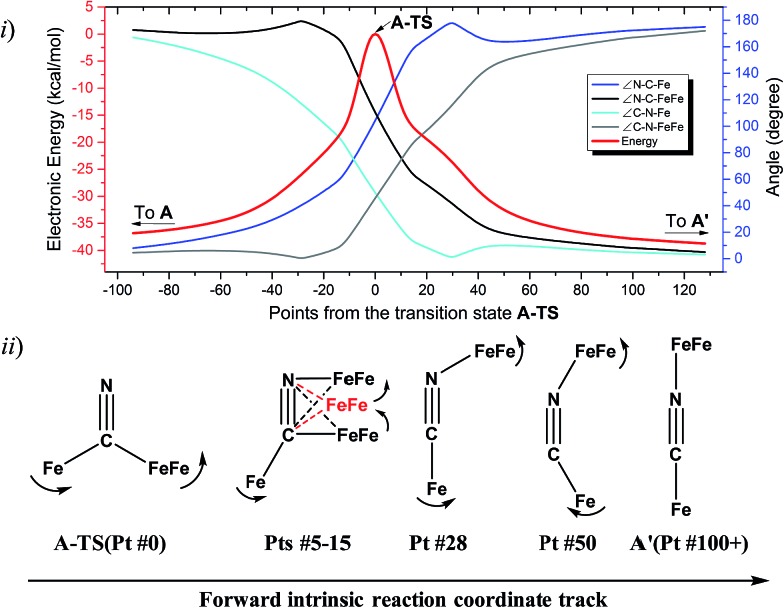
(i) Electronic energy and selected metric data plot of the intrinsic reaction coordinate (IRC) connecting **A** and **A′** through **A-TS** (the left (red) *y*-axis is for energy, and the right (blue) *y*-axis is for bond angle). (ii) The geometries of selected points on the IRC; the motions are indicated with arrows. The shifting of the FeFe moiety on CN is reflected by the sketches of Pts #5-15.

The reaction trajectory involving **A-TS** was obtained from intrinsic reaction coordinate (IRC) calculations and is presented in Fig. 5. The reaction coordinate is explained in the following sentences, taking the path from **A-TS** to **A′** as an example. **A-TS** follows the wagging imaginary vibrational motion first until the FeFe moiety approaches the nitrogen (Pts #5-15). In this manner the FeFe moiety gradually shifts from C to N and ultimately the N–C–Fe angle continues to increase until it is completely linear (Pt #28). After that, the bridging cyanide continues to rotate to align the C–N–FeFe angle and the N–C–Fe re-bends slightly as a side effect until it reaches Pt #50. Finally, both N–C–Fe and C–N–FeFe angles adjust to completely linear as the energy ultimately drops to the local minimum **A′**. As the reaction coordinate is traced downhill, the C–N–FeFe angle increases monotonically. The other branch of the reaction coordinate, *i.e.* from **TS-A** to **A**, has exactly the same features and the validity of the transition state is confirmed. Such a route is partially related to the mechanism developed for HCN ⇆ CNH isomerization with spectroscopic evidence of the transition state.^25^ A similar transition state **D-TS** for complex **D**, which features more bulky substituents on both iron moieties, was estimated to have an even higher barrier of 51.0 kcal mol^–1^. In **D-TS**, both iron moieties are dragged together by the bridging carbon and severe steric repulsion develops. Other trials to locate transition states, including a N-bridged **A-TS′** or a side-on/η^2^-bridged **A-TS′′** of complex **A**, were attempted but yielded higher barriers, *G* = 43.0 and 46.7 kcal mol^–1^, respectively (Fig. 4ii).

A recent paper^26^ expounded on the fact that the nitrogen end of the cyanide anion has stronger affinity to the H-bond than the carbon end. Thus an interesting question is whether properly arranged H-bond providers can compensate for the loss of the N–M bond and facilitate flipping. Thus, we tested the effects of one or two waters, one urea and one protonated pyridine as H-bond providers. However, none of them significantly stabilize the transition state (Table S2†).

To assess the possibilities of intermolecular mechanisms, the isomerization of fragment **1** (Chart 1) in complex **A** was first evaluated computationally. The isomerized species **1′**, with a N-bound terminal cyanide, is 14.6 kcal mol^–1^ less stable than **1**. The barrier is 28.2 kcal mol^–1^ and the transition state **1-TS** features a side-on cyanide. On top of that, it takes 24.4 kcal mol^–1^ to dissociate fragment **1** from **A** (Fig. 4). Alternatively, a (CO)_2_Fe(NC) fragment (**z′**) may be cleaved from complex **A**. The barrier is 14.1 kcal mol^–1^ for **z′** to isomerize into fragment **z** (Chart 1), which is further stabilized by 15.9 kcal mol^–1^ compared to **z′**. Such an isomerization is also overwhelmed by the bond rupture energy of 28.4 kcal mol^–1^ (Fig. 4). Therefore, the intermolecular mechanisms are actually more difficult to access than the intramolecular one presented above.

### Disruption of the cyanide-bridged adducts

**Fig. 6:**
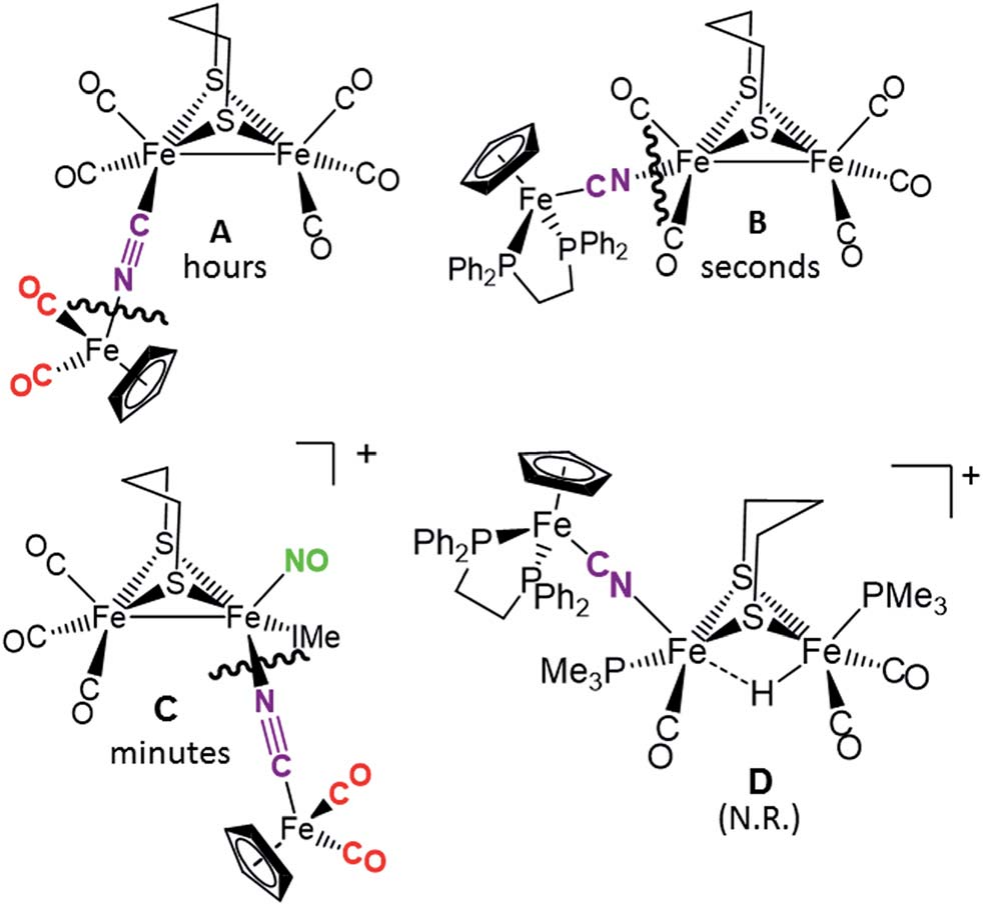
Depiction of the cleavage site of the four bridging cyanide complexes with one equivalent of a strong nucleophile (such as PMe_3_) and the relative time to completion.

Fig. 6 summarizes the results of exposure of the four μ-CN tri-iron complexes to PMe_3_, in possible mimicry of the release or transfer of the di-iron unit from the 4Fe4S cluster of “loaded” HydF, see Fig. 1. All reactions were carried out with a 1 : 1 ratio of phosphine ligand in THF solvent at 22 °C; the time required for complete reaction correlates with expectations for the strength of the CN–Fe interaction. No reaction was observed for what is expected to be the tightest adduct **D**. In the cases of adducts **A**, **B** and **C**, cleavage of the cyanide bridge occurred at the CN–Fe dative bond.

### Comments

Examples of the two possible orientations Fe^II^–NC–[Fe^I^Fe^I^] *vs.* Fe^II^–CN–[Fe^I^Fe^I^] are readily obtained by judicious choice of metallocyanide donor “ligand” with an electrostatically-matched iron receiver. In no case did the orientation of the Fe–CN–Fe′ unit differ from the original components; *i.e.*, as described by Vahrenkamp, the specific linkage isomer is a kinetic isomer, predetermined by the precursors.^13^ Thus, the application of these results to the synthesis of the bio-hybrid *via* HydF^2^ and other CN-bridged polymetallics^11^,^12^ where such cyanide flipping has been observed raises questions. For example how do successive isomerizations take place in the molecular analogues of Prussian blues where three CN^–^ are flipped per M···M′ interaction in the arrangement of M_2_M′_3_ systems^12^?

The MCN···M′ interactions that are readily derived in the organometallic cyanides in our study and in that of Zhu and Vahrenkamp^13^ show strength and stability that relate to the electrostatic differences in the donor/acceptor precursors. Neither cyanide transfer nor flipping was observed in our biomimetic studies of M–CN···M′ adduct formation. Obviously, our studies were carried out in organic solvents wherein the specific H-bonding capabilities and complexity of a protein matrix are missing. The recent description of rigidity of the 2Fe subsite cavity in apo-HydA that is preserved after the enclosure of the synthetic 2Fe model corroborates the need for a specific matrix that adapts the symmetrical synthetic di-iron complex into a structure that is efficient for catalysis.^5^ Such outer sphere effects on the inner coordination sphere structure of a di-iron complex have yet to be imitated.

Based on the biomimetic study results, the energetic preference for the linkage isomers in the protein cavity-enclosed cases and the reported cyanide flipping are still perplexing. Might there be an alternative interpretation of the link between the 4Fe4S cluster and the 2Fe synthetic unit in the hybrid form of HydF? As there has been no crystal structure of this assembly (reported as of now) more detailed EPR experiments are needed both on the bio-hybrids and on faithful, well characterized models.

While such questions persist, it is clear that the qualities that cyanide offers as a bridging ligand well suit them as docking agents for broad applications in bioinorganic chemistry as has been found in materials synthesis. The results above demonstrate the feasibility of stable small molecule paradigms expected for the docking of synthetic di-iron cyano complexes into a protein cavity containing an Fe receiver group. Potential roles of the cyanide ligand in the H-cluster of [FeFe]-H_2_ase now include not only the stabilization of multiple redox levels, and tuning the basicity of the pendant amine, but additionally CN^–^ is a group for directing pre-formed metal fragments into proteins.

## Experimental section

### General

All reactions and manipulations were performed using standard Schlenk-line and syringe/rubber septa techniques under N_2_ or in an Ar atmosphere glovebox. Solvents were purified and degassed *via* a Bruker solvent system. Reagents were purchased from commercial sources and used as received. The known compounds, [(η-C_5_H_5_)Fe(CO)_2_THF][BF_4_],^27^ (η-C_5_H_5_)Fe(CO)_2_CN,^28^ (η-C_5_H_5_)Fe(dppe)CN,^29^ (μ-pdt)[Fe(CO)_3_][Fe(NO)(IMe)(CO)][BF_4_],^20^ (μ-pdt)[Fe_2_(CO)_5_CN][Na],^30^ (μ-pdt)(μ-H)[Fe(CO)_2_PMe_3_]_2_[PF_6_],^21^ NaBArF,^31^ [(η-C_5_H_5_)Fe(CO)_2_PMe_3_][BF_4_],^32^ and (μ-pdt)[Fe_2_(CO)_5_PMe_3_]^33^ were synthesized according to literature procedures.

### Physical measurements

Elemental analyses were performed by Atlantic Microlab, inc., Norcross, Georgia, United States. Electrospray ionization mass spectrometry (ESI-MS) was performed by the Laboratory for Biological Mass Spectrometry at Texas A&M University. Infrared spectra were recorded on a Bruker Tensor 37 spectrometer using a CaF_2_ solution cell of 0.2 mm path length. A Bioanalytical Systems 100 electrochemical workstation with a glassy carbon working electrode (0.071 cm^2^) and a platinum wire auxiliary electrode was used to conduct the electrochemical analysis of all compounds. A standard three electrode cell under an Ar atmosphere at room temperature was used to obtain all voltammograms. Cyclic voltammograms of all complexes as well as starting materials were recorded in 2 mM DCM solutions with 100 mM [*n*-Bu_4_N][PF_6_] as the supporting electrolyte. The potentials were measured relative to a Ag/AgNO_3_ electrode using a glassy carbon working electrode, and are referenced to Cp_2_Fe/Cp_2_Fe^+^ (*E*_1/2_ = 0.00 V). ^1^H and ^31^P NMR spectra were recorded on an Inova 400 MHz superconducting NMR instrument operating at 400 MHz and 161.92 MHz, respectively. ^1^H spectra were referenced to residual solvent while ^31^P spectra were referenced to external H_3_PO_4_. CW EPR spectrum were recorded using an X-Band Bruker 300E spectrometer equipped with an Oxford Instruments helium flow cryostat (Oxford Instr. ESR910) and an ITC 503 temperature controller. Measurement conditions: microwave frequency: 9.643 GHz; modulation amplitude: 0.3 mT; microwave power: 0.04 mW; temperature: 10 K. Coulometric oxidation was performed in a glovebox at –35 °C with an EG & G Potentiostat 273A, a Ag/AgNO_3_ reference electrode in CH_3_CN, a Pt-wire counter electrode, and a Pt-mesh working electrode. The solution was transferred to an EPR tube, removed from the glovebox, and immediately frozen in liquid N_2_. A cyclic voltammogram was performed before and after the coulometric oxidation and gave similar features in both cases. The similarity of cyclic voltammograms of complex **D** performed before and after the coulometric oxidation indicated the preservation of the structure in the **D**/**D^+^** conversion.

### X-ray structure analysis

Low temperature (150 K) X-ray data were obtained on a Bruker Apex-II CCD based diffractometer (Texas A & M University) (Mo sealed X-ray tube, K_α_ = 0.71073 Å) for complexes **A–D**. Crystal samples were coated in mineral oil, affixed to a Nylon loop, and placed under streaming N_2_. Space groups were determined on the basis of systematic absences and intensity statistics, and structures were solved by direct methods and refined by full-matrix least-squares on F_2_. All non-hydrogen atoms were refined with anisotropic thermal parameters. H atoms were placed at idealized positions and refined with fixed isotropic displacement parameters; anisotropic displacement parameters were employed for all non-hydrogen atoms. The following programs were used: data collection, APEX2;^34^ data reduction, SAINT;^35^ absorption correction SADABS;^36^ cell refinement SHELXTL;^36^ structure solutions, SHELXS-97;^36^ and structure refinement, SHELXL-97.^36^ The final data presentation and structure plots were generated in X-Seed Version 2.0.^37^

### Computational methodology

Gaussian 09 Version D01 (ref. 38) was used to execute all the calculations unless noted otherwise. The TPSS functional^39^ was applied for most calculations except EPR parameter determination, in which case the functional B3LYP^40^ was utilized. Triple-*ζ* 6-311++G(d,p)^41^–^43^ basis set was used for all non-metal atoms, except the methyl and phenyl groups on the phosphines in complexes **B** and **D**, for which double-*ζ* 6-31G basis set^44^,^45^ was used to save computational resources. For 1^st^ row transition metal Fe, Wachters-Hay basis set with diffuse functions and polarization functions was applied, under the designation 6-311++G(d,p).^46^–^48^ The crystal geometries of **A–D** determined from X-ray diffraction were used as initial guesses in the optimizations. The unobserved CN-linkage isomers were generated by manually exchanging the positions of N and C. The initial structures of possible transition states were generated by relaxed scans with educated guesses. All the geometries were optimized to stationary points in a vacuum and further verified by the existence of proper numbers of imaginary vibrational frequencies, *i.e.* zero for a minimum and one for a transition state. Thermal corrections from vibrational calculations, along with solvation corrections in acetonitrile using the SMD model,^49^ were added to calculate the Gibbs free energies in solution. Grimme's empirical dispersion GD3BJ^50^,^51^ was only added to the evaluation of H-bond stabilization (Table S2†). The Intrinsic Reaction Coordinate was tracked by following the normal mode related to the imaginary vibrational frequency of the transition state, given by a normalized Hessian matrix. The Hessian matrix was updated every ten steps and the track stopped till a minimum was located on the electronic potential surface. The EPR parameters were calculated using ORCA^52^ with the functional B3LYP.^40^ The *g* tensors and *A* tensors were calculated based on the optimized geometries extracted from Gaussian. The *A* tensors include both the dipole part and the second order contribution part of spin–orbital coupling. These tensors were diagonalized to generate eigenvalues.

### Synthesis of compound **A**

A dark red solution of [Na][(μ-pdt)[Fe_2_(CO)_5_CN] was generated using the published procedure using 0.5 g (μ-pdt)[Fe_2_(CO)_6_] (1.3 mmol) and 0.13 mL of a 1.0 M solution of sodium bis(trimethylsilyl)amide in THF. The resulting solution was transferred to a degassed round bottom flask containing 0.511 g of [(η-C_5_H_5_)Fe(CO)_2_THF][BF_4_] (1.3 mmol). The reaction was allowed to stir until completely converted to the bridging cyanide product as determined using IR (∼30 min). The solvent was removed and the remaining solid was loaded onto a 30 cm silica gel column eluting first with hexane to remove an orange band corresponding to (μ-pdt)[Fe_2_(CO)_6_] followed by a solvent gradient to pure diethyl ether to initially remove any [(η-C_5_H_5_)Fe(CO)_2_]_2_ generated during the reaction. Compound **A** finally elutes followed by solvent removal to produce 0.568 g of compound **A** resulting in a 78% yield of an orange/brown solid. X-ray quality crystals were grown by slow diffusion of hexane into a DCM solution of compound **A** at –30 °C. IR (THF, cm^–1^) *ν*(CO) 2069 (m), 2026 (m), 1986 (s), 1955 (s), 1930 (m); *ν*(CN) 2128 (m). Anal. found (calcd) for Fe_3_S_2_NO_7_C_16_H_11_: C, 34.57 (34.26); H, 2.09 (1.98); N, 2.51 (2.50). ^1^H NMR (400 MHz, CD_3_OD): *δ* 1.5–2.4 (6H, br) 4.89 (5H, S).

### Synthesis of compound **B**

To a Schlenk flask containing 0.25 g (μ-pdt)[Fe_2_(CO)_6_] (0.64 mmol), 0.294 g (η-C_5_H_5_)Fe(dppe)CN (0.54 mmol) and 0.037 g Me_3_NO (0.5 mmol) was added 30 mL of THF and stirred for 2 hours. The resulting dark brown solution was taken to dryness under reduced pressure, dissolved in 3 mL ether and filtered through a plug of Celite under an inert atmosphere. A dark green solid was precipitated with 40 mL hexane followed by washing with hexane until the supernatant was no longer orange. The solid was dried under vacuum yielding 0.336 g of compound **B** corresponding to a 69% yield. X-ray quality crystals were grown by slow evaporation of a 5 : 1 DCM : hexane solution at –30 °C. IR (THF, cm^–1^) *ν*(CO) 2035 (m), 2028 (m), 1980 (s), 1947 (m), 1919 (m); *ν*(CN) 2100 (w). Anal. found (calcd) for Fe_3_P_2_S_2_NO_5_C_40_H_35_: C, 52.96 (53.19); H, 1.56 (1.55); N, 3.94 (3.91). ^1^H NMR (400 MHz, CDCl_3_): *δ* 7.05–7.85 (20H, m) 4.16 (5H, s) 1.97–2.21 (4H, m) 1.25–1.8 (6H, m). ^31^P NMR (400 MHz, CD_2_Cl_2_): *δ* 102.5–104.2 (CH_2_**P**Ph, m).

### Synthesis of compound **C**

To a Schlenk flask containing 0.184 g (μ-pdt)[Fe(CO)_3_][Fe(NO)(IMe)(CO)][BF_4_] (0.34 mmol) dissolved in 30 mL of DCM at 0 °C was added 0.1 g (η-C_5_H_5_)Fe(CO)_2_CN (0.51 mmol) under a back pressure of N_2_. The solution was stirred for 30 min and allowed to warm to room temperature followed by filtration through a plug of Celite. The resulting solution was loaded onto a 30 cm silica gel column and eluted with 50% THF/DCM to elute a yellow band of (η-C_5_H_5_)Fe(CO)_2_CN followed by a red band of compound **C**. Removal of the solvent resulted in 0.166 g of a red solid in a 67% yield. X-ray quality crystals were grown by slow evaporation of a DCM solution at room temperature. IR (DCM, cm^–1^) *ν*(CO) 2067 (m), 2060 (m), 2024 (s), 1995 (m); *ν*(CN) 2154 (w); *ν*(NO) 1755 (m). Anal. found (calcd) for Fe_3_S_2_N_4_O_6_C_19_H_20_BF_4_: C, 32.04 (31.79); H, 2.75 (2.67); N, 7.55 (7.81). ESI-MS^+^ (CH_2_Cl_2_): *m*/*z* = 630.85 (μ-pdt) [CpFe(CO)_2_CN] [FeNO(IMe)] [Fe(CO)_3_]^+^. ^1^H NMR (400 MHz, CD_2_Cl_2_): *δ* 7.17 (2H, s) 5.22 (5H, s) 3.78 (6H, s) 1.2–2.1 (6H, m).

### Synthesis of compound **D**

A Schlenk flask was loaded with 0.095 g solid (η-C_5_H_5_)Fe(dppe)CN (0.17 mmol) and 0.1 g (μ-pdt)(μ-H)[Fe(CO)_2_PMe_3_]_2_[PF_6_] (0.16 mmol) and purged with Ar for 15 min followed by the addition of 30 mL of DCM. The flask was cooled to 0 °C and illuminated with a 275 W GE ultraviolet sunlamp for 6 h. Due to the heat produced by the sun lamp, care was taken to ensure the solution is kept at 0 °C. The sunlamp was removed, the solution stirred at room temperature for 8 h and loaded onto a 30 cm column eluting with 5% THF in DCM. The dark green band was collected and the solvent removed to afford 0.107 g of compound **C** in a 58% yield. IR (DCM, cm^–1^) *ν*(CO) 2020 (m), 1964 (m), 1949 (s); *ν*(CN) 2081 (w). Anal. found (calcd) for Fe_3_S_2_P_5_NO_3_C_44_F_6_H_54_·OC_4_H_10_ C, 47.46 (47.27); H, 5.19 (5.29); N, 1.22 (1.15). ESI-MS^+^ (CH_2_Cl_2_): *m*/*z* = 1000.11 (μ-pdt)(μ-H)[CpFe(dppe)CN][Fe(CO)PMe_3_][Fe(CO)_2_PMe_3_]^+^. ^1^H NMR (400 MHz, CD_2_Cl_2_): *δ* –27.35 (1H, dd) –10.54 (1H, dd) 1.1–1.4 (18H, m) 1.5–2.9 (10H, m) 4.39 (5H, t) 6.9–8.0 (20H, m). ^31^P NMR (400 MHz, CDCl_3_): *δ* –145.0 (**P**F_6_, sept) 21.5–28.0 (**P**Me_3_, m) 101.0–103.0 (CH_2_**P**Ph, m). X-ray quality crystals were grown by first conducting a counter ion exchange with NaBArF. Compound **D** (0.1 g, 0.087 mmol) and 0.071 g NaBArF (0.08 mmol) were dissolved in 5 mL DCM in a Schlenk flask and stirred overnight. The solvent was removed and the residue dissolved in 15 mL diethyl ether, filtered through a plug of Celite and the solvent removed to afford 0.143 g of compound. Crystals were grown by slow diffusion of hexane into a solution of **D** in THF.

### General procedure for the cleavage reactions using trimethylphosphine

To a 5 mL vial containing 0.05 mmol of the appropriate bridging cyanide compound and a stir bar was added 1 mL of a 0.05 mM solution of PMe_3_ dissolved in THF. The resulting solution was stirred and monitored using IR spectroscopy until the reaction was judged to be complete by the disappearance of the bridging cyanide peak. Compound **D** showed no reaction with PMe_3_ even at elevated temperatures or increased PMe_3_ concentration.

#### Cleavage of compound **A**

The reaction was judged to be complete after 6 h by the disappearance of the IR stretch at 2128 cm^–1^ corresponding to the bridging cyanide. Fig. S13† shows the new peaks which formed and the assignment of these stretches.

#### Cleavage of compound **B**

The reaction was complete upon adding the PMe_3_ evident by the immediate color change from green/brown to red. Fig. S15† shows the new peaks which formed and the assignment of these stretches.

#### Cleavage of compound **C**

The reaction was complete after 30 minutes evident by the disappearance of the IR stretch at 2154 cm^–1^ corresponding to the bridging cyanide. Fig. S17† shows the new peaks which formed and the assignment of these stretches.

### General procedure for the cleavage reactions using tetraethylammonium cyanide

To a 5 mL vial containing 0.05 mmol of the appropriate bridging cyanide compound, 1 equiv. of [NEt_4_][CN] and a stir bar were added along with 3 mL of THF. The resulting solution was stirred and monitored using IR spectroscopy until the reaction was judged to be complete by the disappearance of the bridging cyanide peak. Compound **D** showed no reaction with [NEt_4_][CN] even at elevated temperatures or increased concentrations of [NEt_4_][CN].

#### Cleavage of compound **A**

The reaction was judged to be complete after 4 h by the disappearance of the IR stretch at 2128 cm^–1^ corresponding to the bridging cyanide. Fig. S14† shows the new peaks which formed and the assignment of these stretches.

#### Cleavage of compound **B**

The reaction was complete upon adding the [NEt_4_][CN] evident by the immediate color change from green/brown to red. Fig. S16† shows the new peaks which formed and the assignment of these stretches.

#### Cleavage of compound **C**

The reaction was judged to be complete after 45 min by the disappearance of the IR stretch at 2154 cm^–1^ corresponding to the bridging cyanide. Fig. S18† shows the new peaks which formed and the assignment of these stretches.

## Conflict of interest

The authors declare no competing financial interest.

## Supplementary Material

Supplementary information

Supplementary information

Crystal structure data
