# Supplementary material for: Cyanide-bridged iron complexes as biomimetics of tri-iron arrangements in maturases of the H cluster of the di-iron hydrogenase
Source: Chem Sci. 2016 Feb 29;7(6):3710–9. doi: 10.1039/c6sc00213g (PMC6008931; doi:10.1039/c6sc00213g)
Supplement: Supplementary file 1 [file SC-007-C6SC00213G-s001.pdf]

Electronic Supplementary Material (ESI) for Chemical Science.
This journal is © The Royal Society of Chemistry 2016

**Supplemental Information**

# Cyanide-Bridged Iron Complexes as Biomimetics of Tri-iron Arrangements in Maturases of the H cluster of the Diiron Hydrogenase

Allen M. Lunsford[†], Christopher C. Beto[†a], Shengda Ding[†], Özlen F. Erdem[δ], Ning Wang[†b], Nattamai Bhuvanesh[†], Michael B. Hall[†] and Marcetta Y. Darensbourg[†]*

[†]*Department of Chemistry, Texas A & M University, College Station, TX 77843, United States*

[δ]*Department of Physics, Middle East Technical University, 06800 Ankara, Turkey*

E-mail: marcetta@chem.tamu.edu

## Cyclic Voltammetry

### Full Scans

**Figure S1.** Full scan of Compound **A** ....................................................................................................4
**Figure S2.** Full scan of Compound **B**....................................................................................................5
**Figure S3.** Full scan of Compound **C** ....................................................................................................6
**Figure S4.** Full scan of Compound **D** ....................................................................................................7

### Variable Scan Rates

**Figure S5.** Full scan of Compound **A** at increasing scan rates ..........................................................8
**Figure S6.** Cathodic region of Compound **C** at increasing scan rates ...............................................9
**Figure S7.** Full scan of Compound **D** at increasing scan rates ..........................................................10

### Scan Reversals

**Figure S8.** Scan reversals of compound **A** ..........................................................................................11
**Figure S9.** Full scan of Compound **B** initiating the scan in the negative and positive direction ...............12
**Figure S10.** Full scan of Compound **C** initiating the scan in the negative and positive direction..............13
**Figure S11.** Full scan of Compound **D** initiating the scan in the negative and positive direction..............14

### IR Spectrum of Compounds A-D and Cleavage Reactions

**Figure S12.** Stacked normalized IR plots of Compounds **A-D** in DCM.................................................15
**Figure S13.** IR spectrum of the cleavage of Compound **A** using $PMe_3$.................................................16
**Figure S14.** IR spectrum of the cleavage of Compound **A** using tetraethylammonium cyanide...............17
**Figure S15.** IR spectrum of the cleavage of Compound **B** using $PMe_3$**.**................................................18
**Figure S16.** IR spectrum of the cleavage of Compound **B** using tetraethylammonium cyanide. ..............19
**Figure S17.** IR spectrum of the cleavage of Compound **C** using $PMe_3$.................................................20
**Figure S18.** IR spectrum of the cleavage of Compound **C** using tetraethylammonium cyanide...............21

### NMRs of Compounds A-D

**Figure S19.** $^{1}$H NMR of Compound **A** in $CD_3OD$ ......................................................................22
**Figure S20.** $^{1}$H NMR of Compound **B** in $CDCl_3$. .......................................................................23
**Figure S21**. $^{31}$P NMR of Compound **B** in $CDCl_3$.........................................................................23
**Figure S22.** $^{1}$H NMR of Compound **C** in $CD_2Cl_2$. ....................................................................24
**Figure S23.** $^{1}$H NMR of Compound **D** in $CD_2Cl_2$......................................................................25
**Figure S24.** $^{31}$P NMR of Compound **D** in $CD_2Cl_2$ ....................................................................26

### EPR

**Figure S25.** The spin density contour plot of oxidized **D**$^{+}$ calculated by B3LYP.. ..................................27
**Figure S26.** X-band 14N HYSCORE spectrum of D$^{+}$...............................................................................28

**Crystallographic Images**

**Figure S27.** Structures for compounds **A-D** as determined by X-ray diffraction experiments with the analogous chemdraw structures........................................................................................................................................29
**Figure S28.** Comparison of the shape of the thermal ellipsoid plots and resulting refinement factor for the two cyanide orientations for Compound **A.**..........................................................................................................30
**Figure S29.** Comparison of the shape of the thermal ellipsoid plots and resulting refinement factor for the two cyanide orientations for Compound **B.**..........................................................................................................30
**Figure S30.** Comparison of the shape of the thermal ellipsoid plots and resulting refinement factor for the two cyanide orientations for Compound **C**...........................................................................................................31
**Figure S31.** Comparison of the shape of the thermal ellipsoid plots and resulting refinement factor for the two cyanide orientations for Compound **D**...........................................................................................................31
**Figure S32.** Thermal ellipsoid plot at 50% probability for **A**. ....................................................................37
**Figure S33.** Thermal ellipsoid plot at 50% probability for **B**. ....................................................................39
**Figure S34.** Thermal ellipsoid plot at 50% probability for **C**. ....................................................................41
**Figure S35.** Thermal ellipsoid plot at 50% probability for **D**. ....................................................................43

**Tables of Bond Distances and Angles Obtained from the Crystal Structures, IR Frequencies, and EPR Parameters**

**Table S1.** Selected metric data of optimized structures in comparison to experimental values. ...............................32
**Table S2.** Computational IR frequencies in comparison with experimental values ....................................................33
**Table S3.** Computational EPR parameters in comparison with experimental values..................................................34
**Table S4.** Energy profiles (Gibbs free energy in kcal/mol) of cyanide flipping with H-bond providers. .................35
**Table S5.** Crystal data and structure refinement for Compound **A**............................................................................36
**Table S6**. Crystal data and structure refinement for Compound **B**. ...........................................................................38
**Table S7.** Crystal data and structure refinement for Compound **C**. ...........................................................................40
**Table S8.** Crystal data and structure refinement for Compound **D**. ...........................................................................42

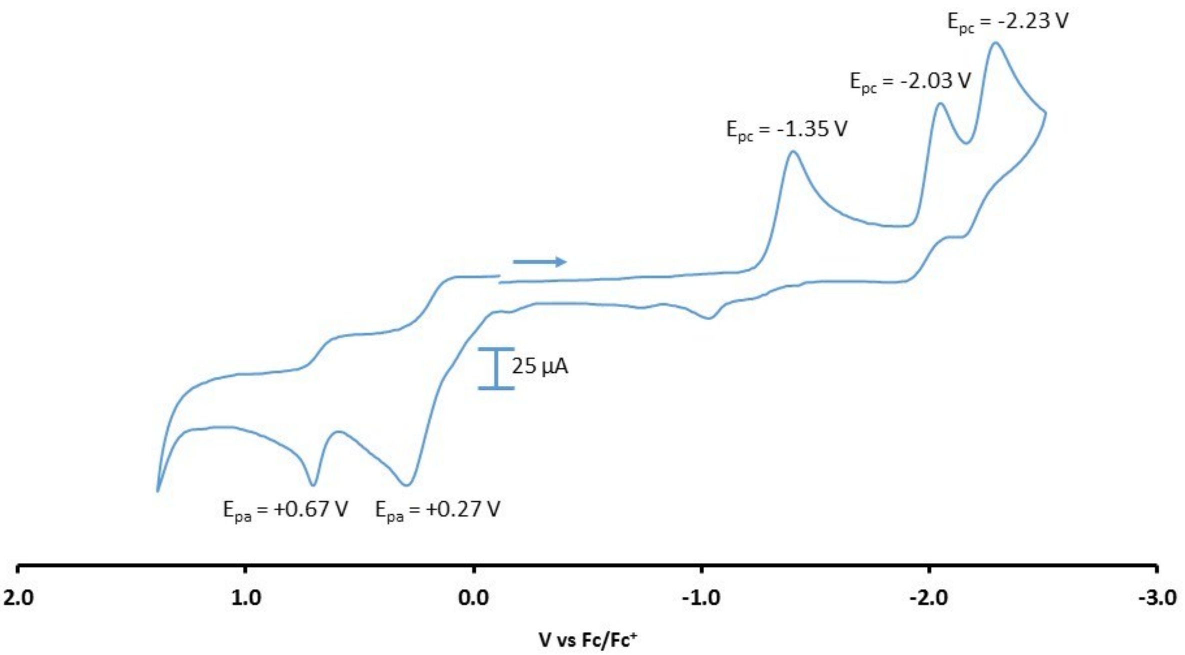


**Figure S1.** Full scan of Compound **A** at 200 mV/s in MeCN referenced to internal $Fc/Fc^{+} = 0$.

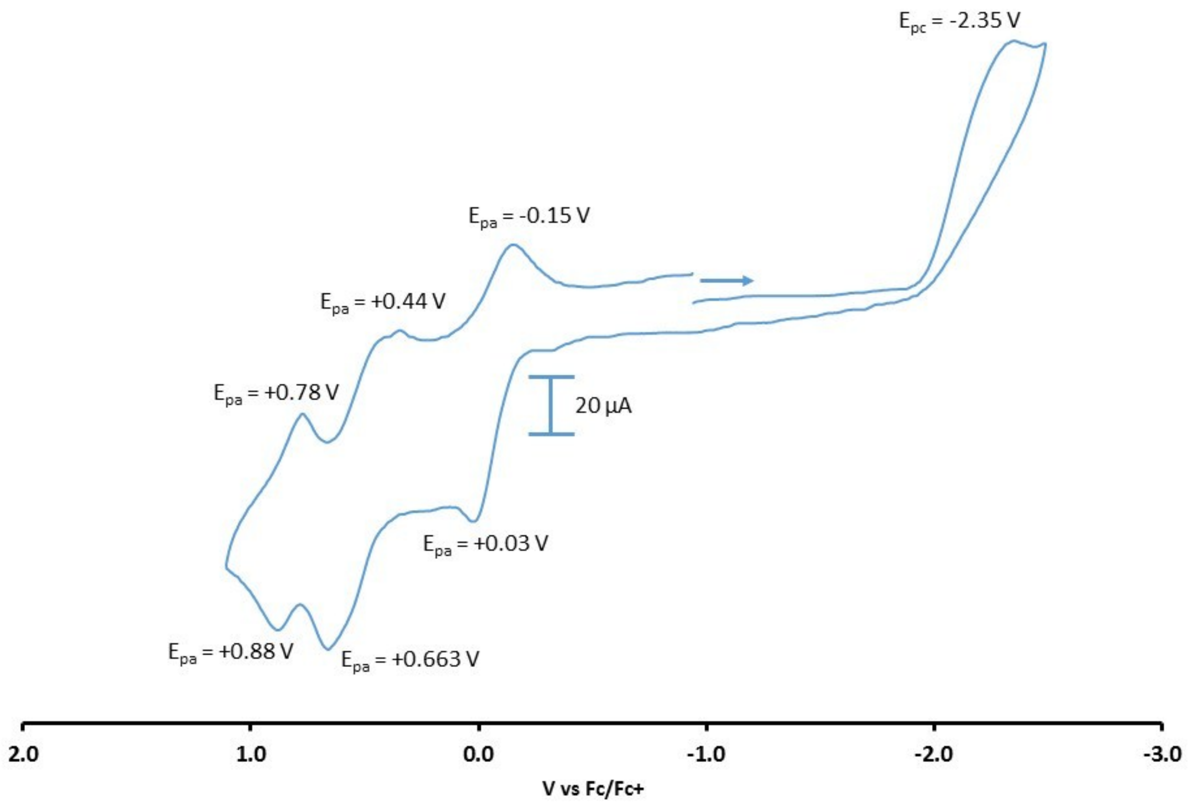


**Figure S2.** Full scan of Compound **B** at 200 mV/s in DCM referenced to internal $Fc/Fc^{+} = 0$.

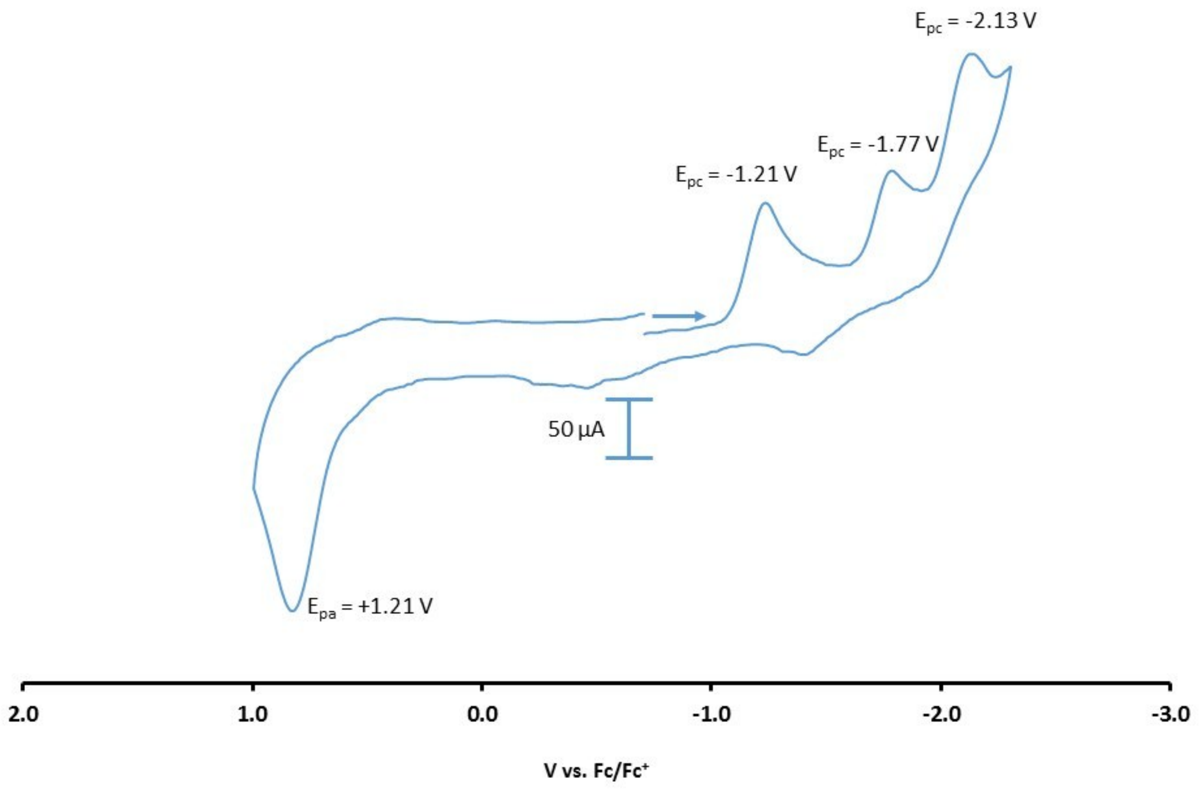


**Figure S3.** Full scan of Compound **C** at 200 mV/s in DCM referenced to internal Fc/Fc$^+$ = 0.

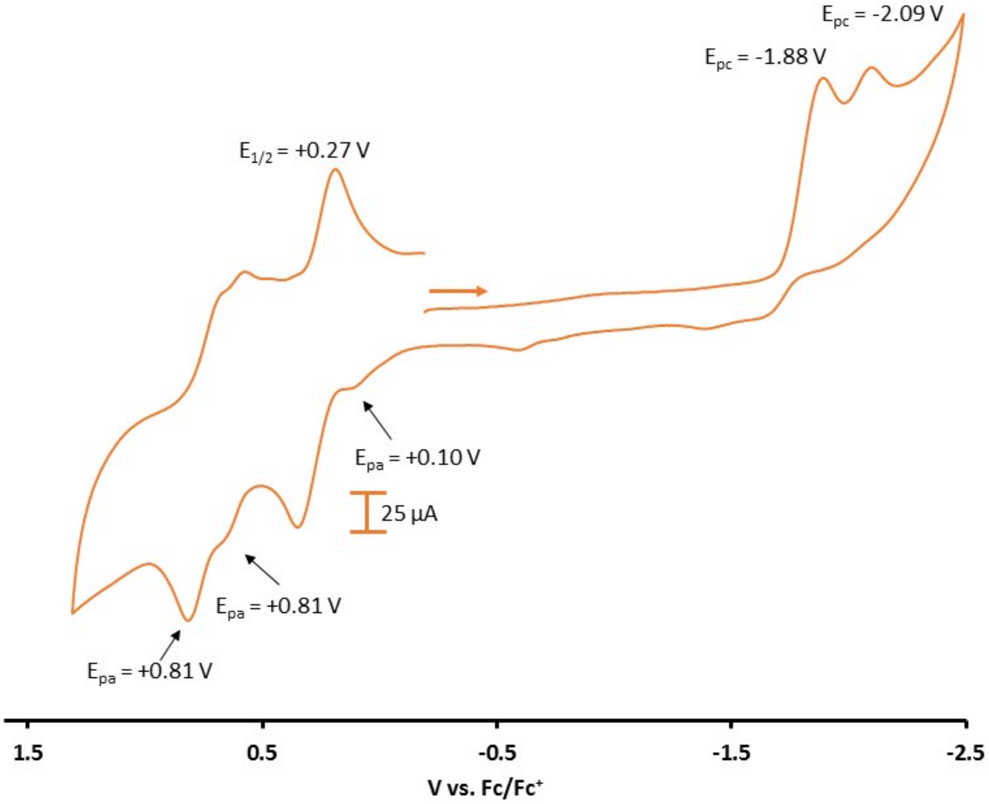


**Figure S4.** Full scan of Compound **D** at 200 mV/s initiating the scan in the negative direction (top) and the positive direction (bottom) in DCM referenced to internal $Fc/Fc^{+}$ = 0.

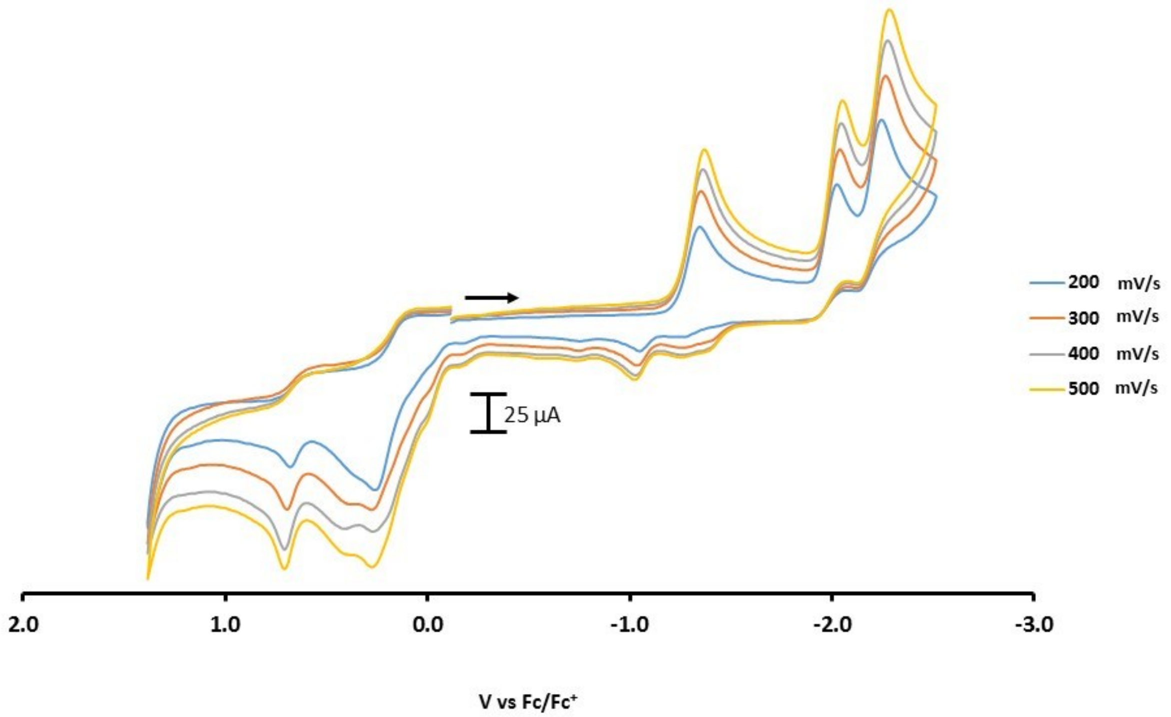


**Figure S5.** Full scan of Compound **A** at increasing scan rates in MeCN referenced to internal $Fc/Fc^{+} = 0$.

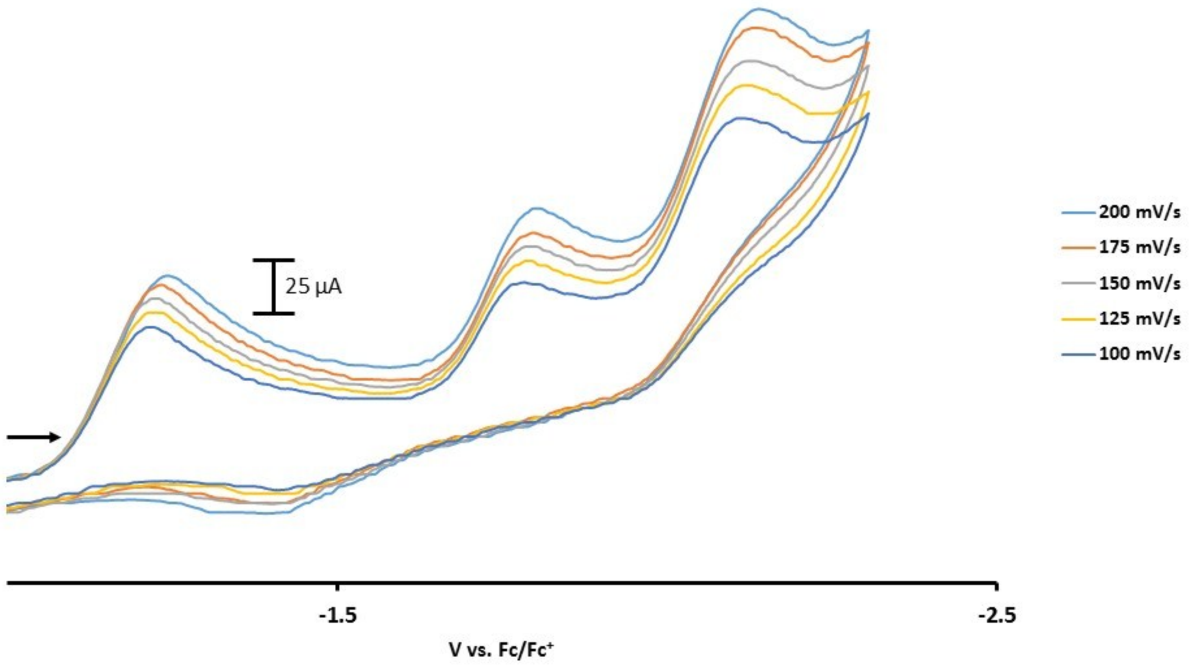


**Figure S6.** Cathodic region of Compound **C** at increasing scan rates in DCM referenced to internal $Fc/Fc^{+} = 0$.

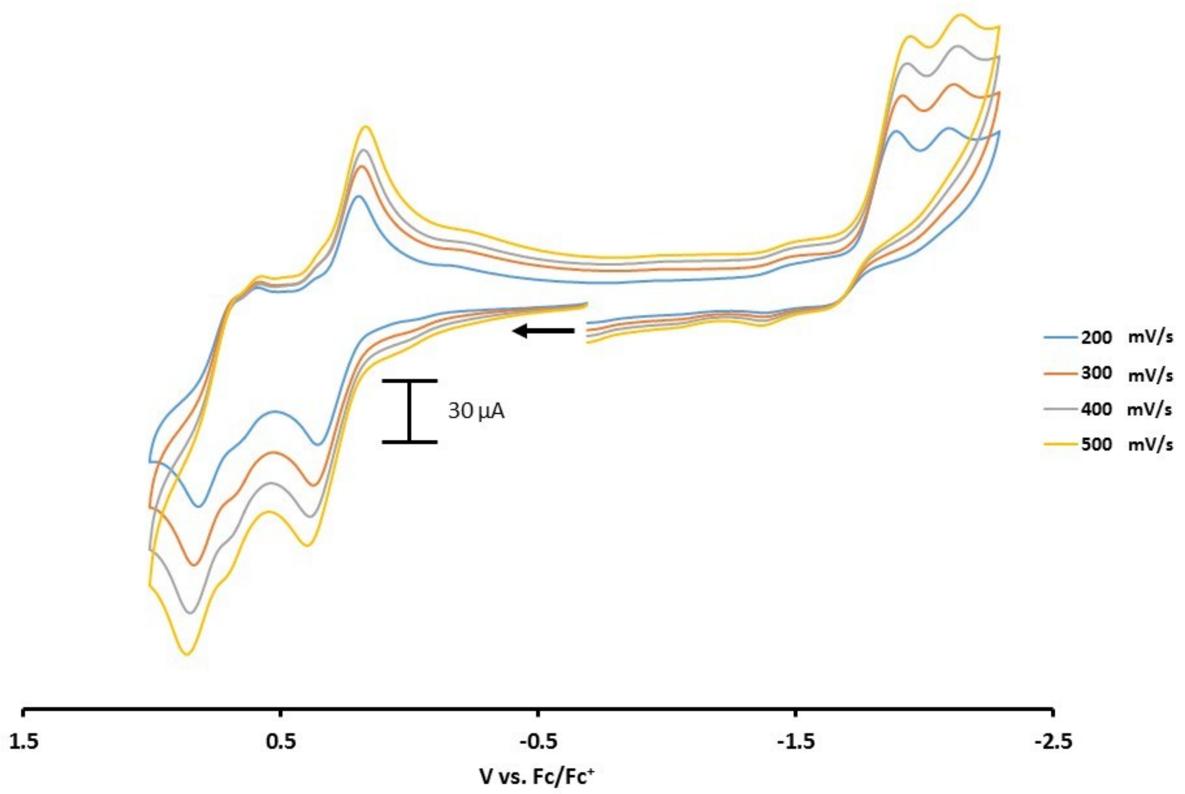


**Figure S7.** Full scan of Compound **D** at increasing scan rates in DCM referenced to internal $Fc/Fc^{+} = 0$.

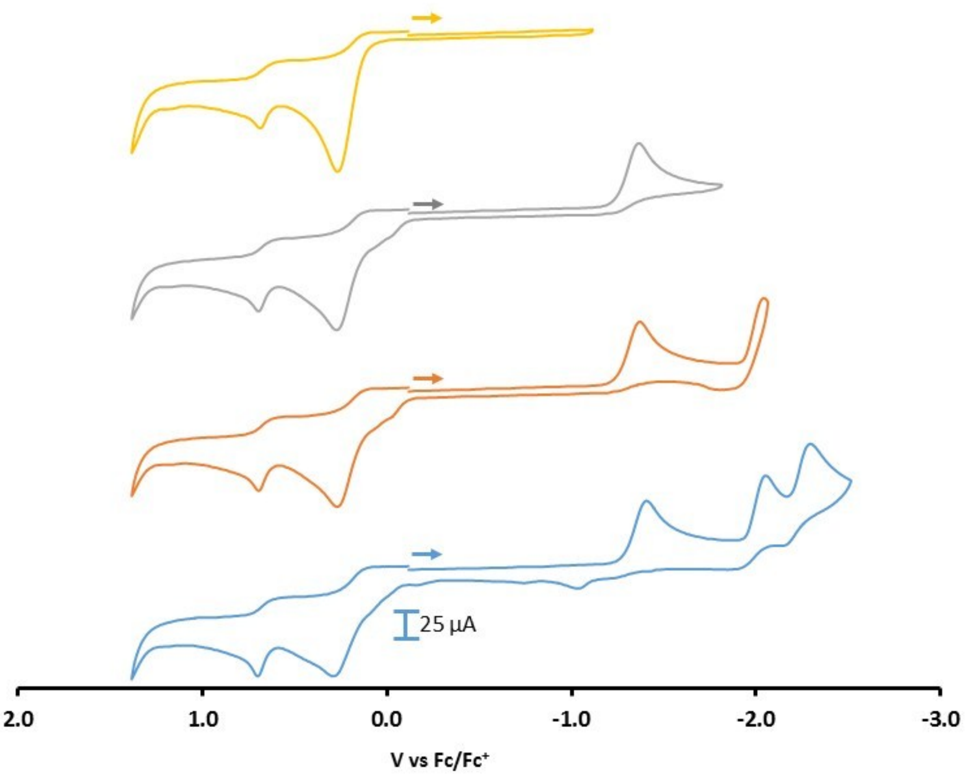


**Figure S8.** Scan reversals of Compound **A** isolating each reduction event and observing the effect on the anodic region in MeCN referenced to internal $Fc/Fc^{+}$ = 0.

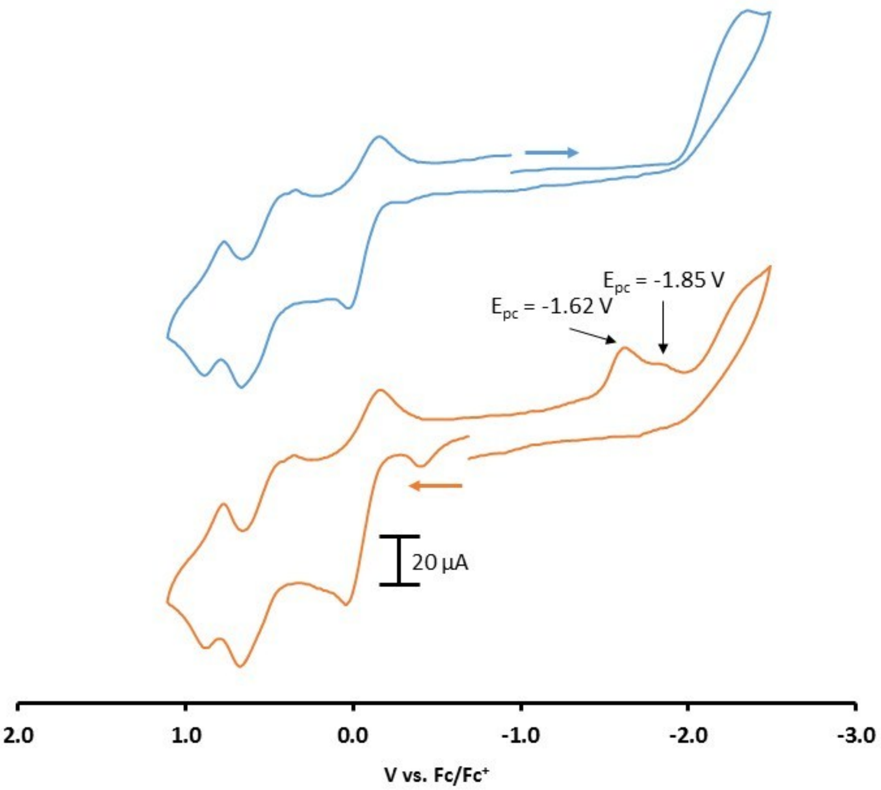


**Figure S9.** Full scan of Compound **B** at 200 mV/s initiating the scan in the negative direction (top) and the positive direction (bottom) in DCM referenced to internal $Fc/Fc^{+} = 0$.

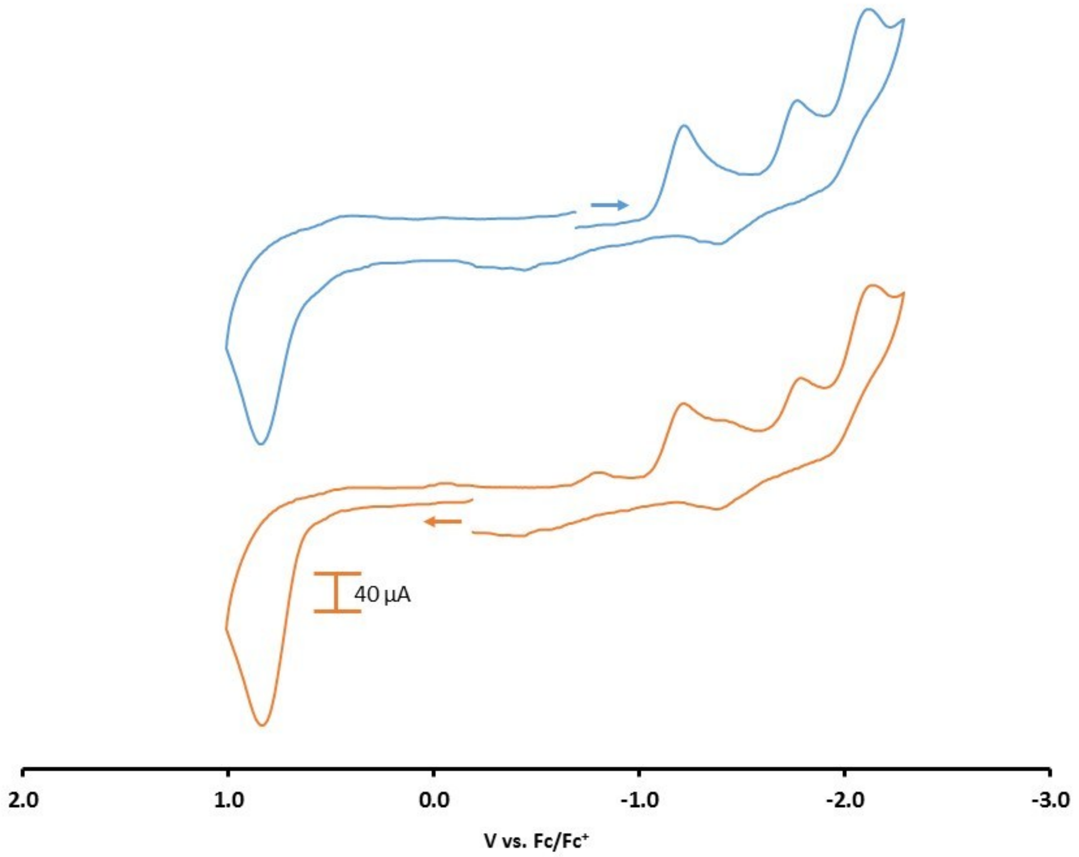


**Figure S10.** Full scan of Compound **C** at 200 mV/s initiating the scan in the negative direction (top) and the positive direction (bottom) in DCM referenced to internal $Fc/Fc^{+} = 0$.

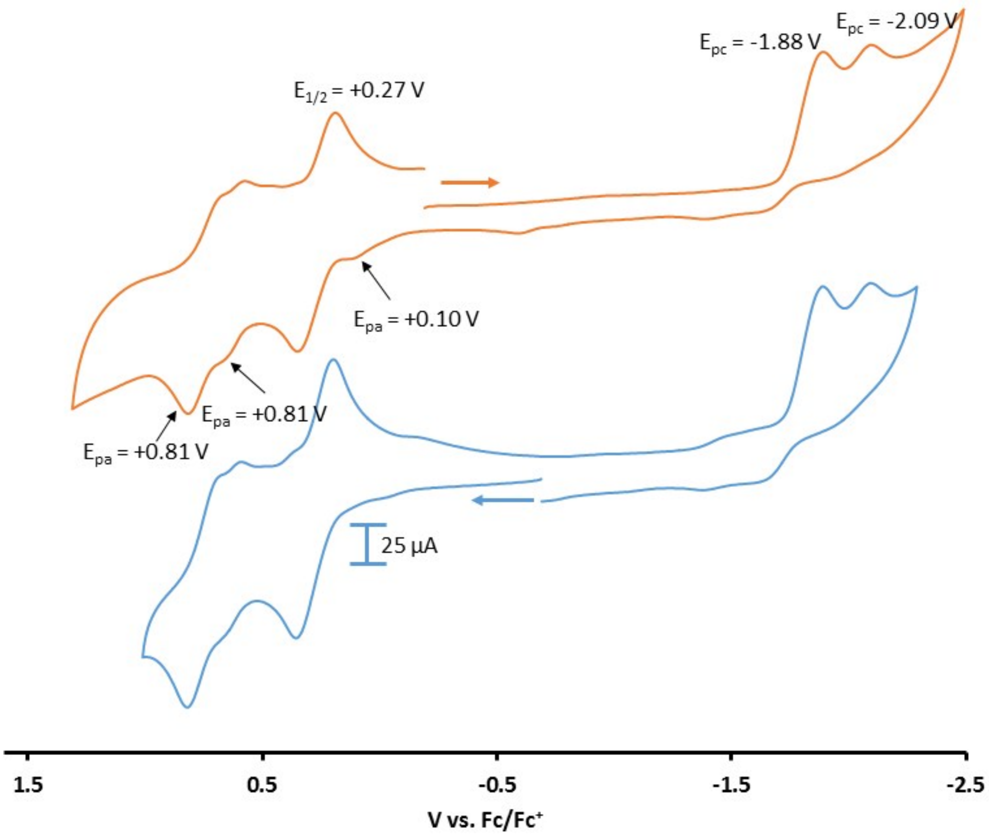


**Figure S11.** Full scan of Compound **D** at 200 mV/s initiating the scan in the negative direction (top) and the positive direction (bottom) in DCM referenced to internal Fc/Fc$^{+}$ = 0.

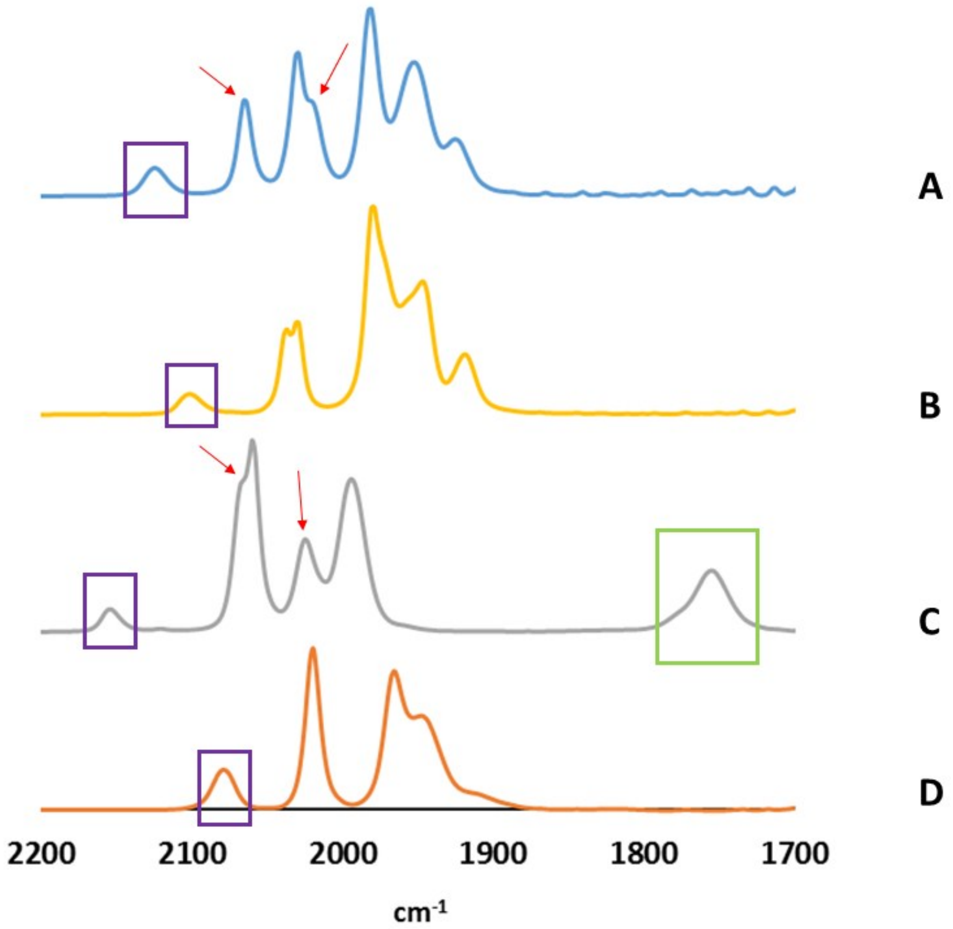


**Figure S12.** Stacked normalized IR plots of Compounds **A-D** in DCM. Red arrows denote CO stretches on the CpFe unit, purple boxes denote bridging cyanide bands, and the green box denotes the NO stretch.

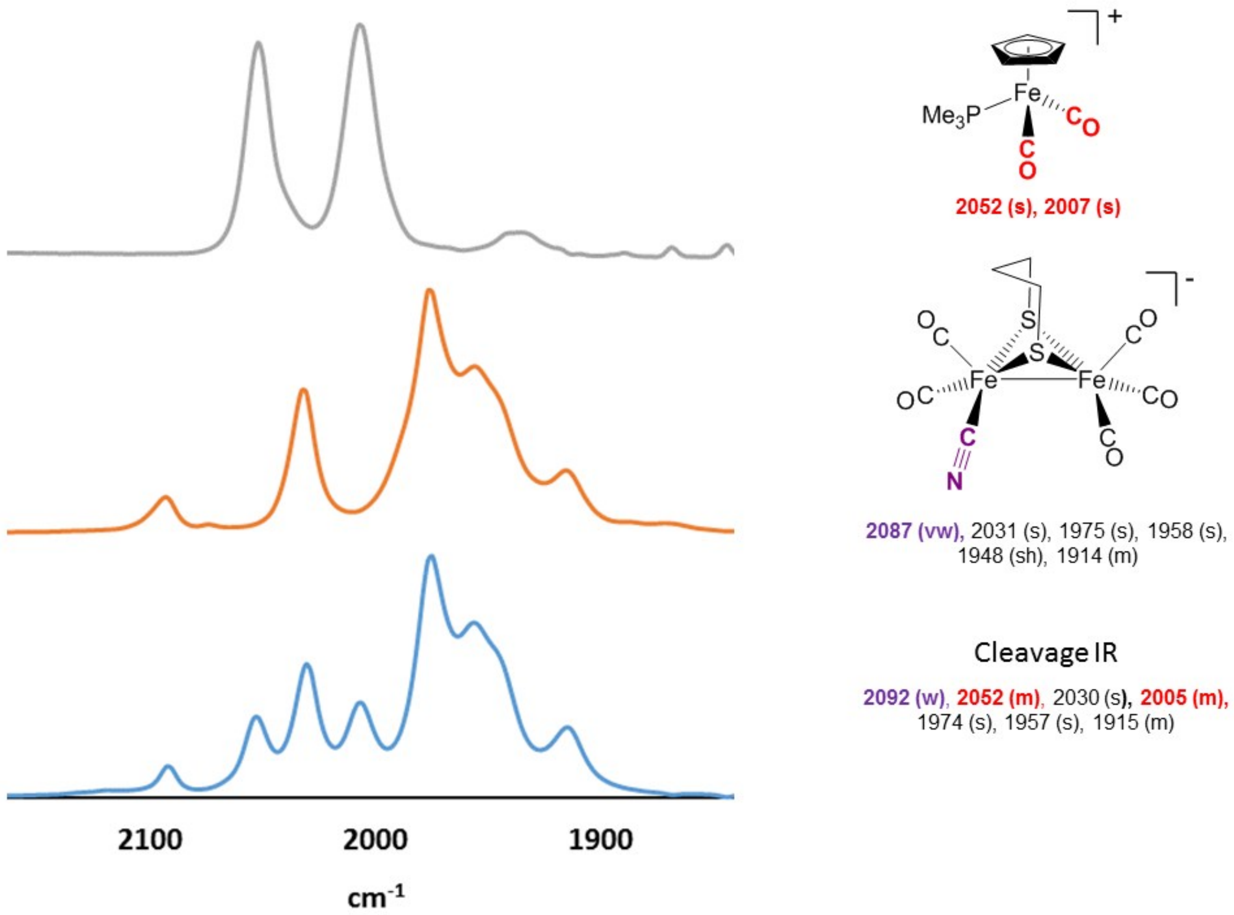


**Figure S13.** IR spectrum in THF of the cleavage of Compound **A** using $PMe_3$.

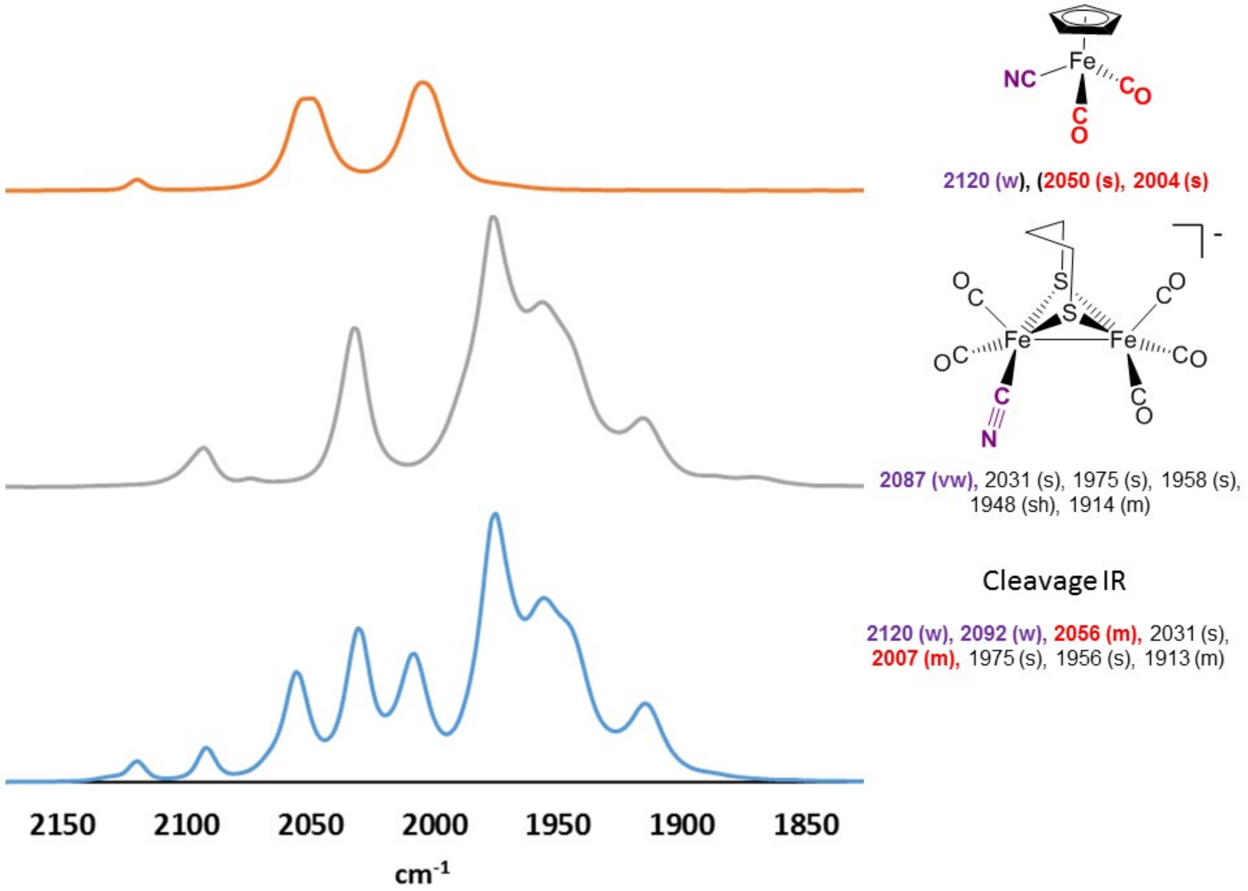


**Figure S14.** IR spectrum in THF of the cleavage of Compound **A** using tetraethylammonium cyanide.

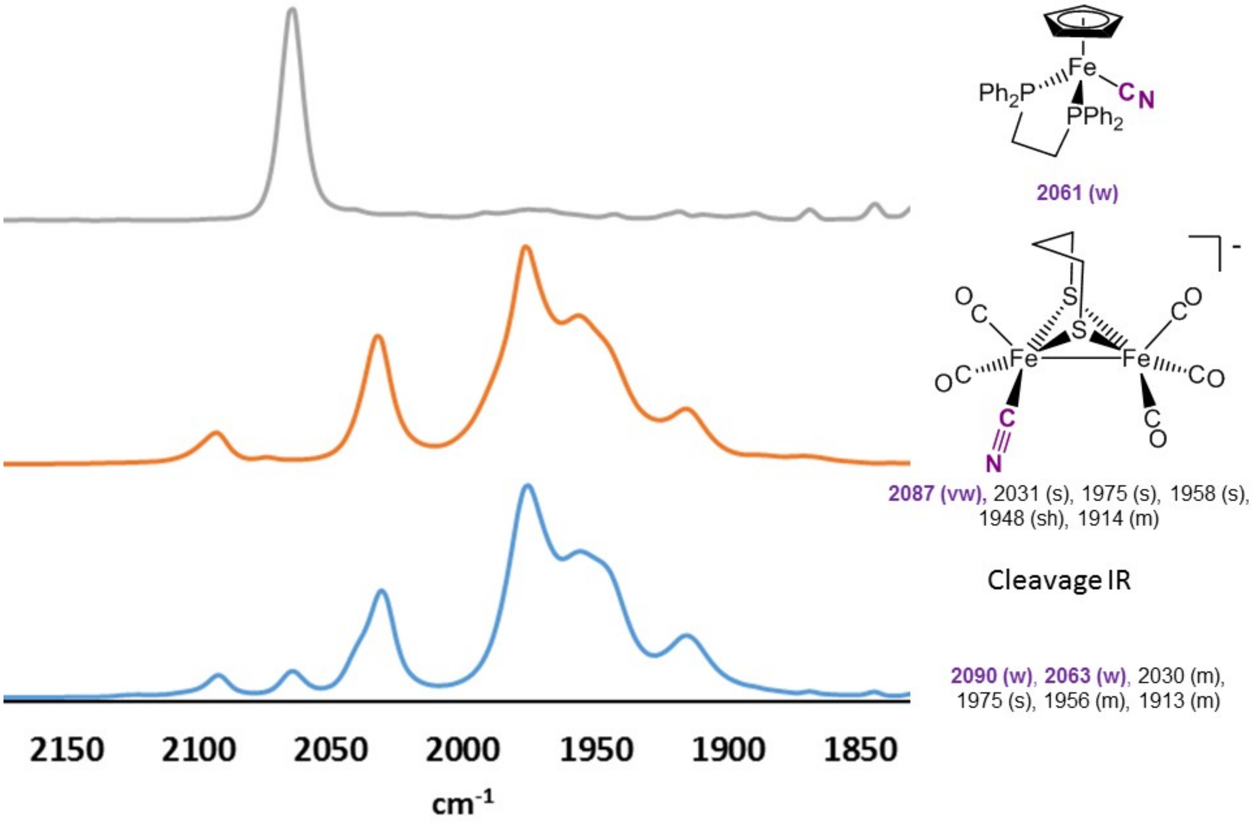


**Figure S15.** IR spectrum in THF of the cleavage of Compound **B** using $PMe_3$.

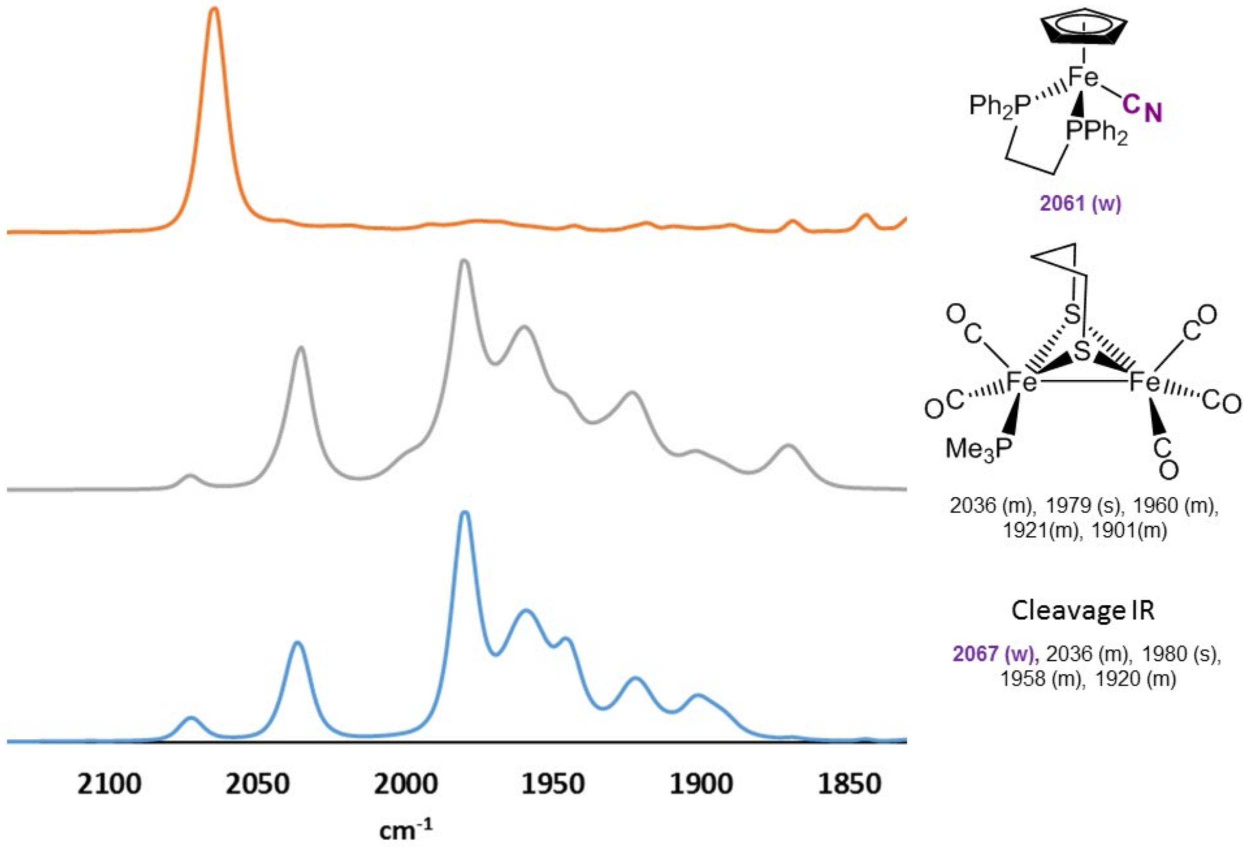


**Figure S16.** IR spectrum in THF of the cleavage of Compound **B** using tetraethylammonium cyanide.

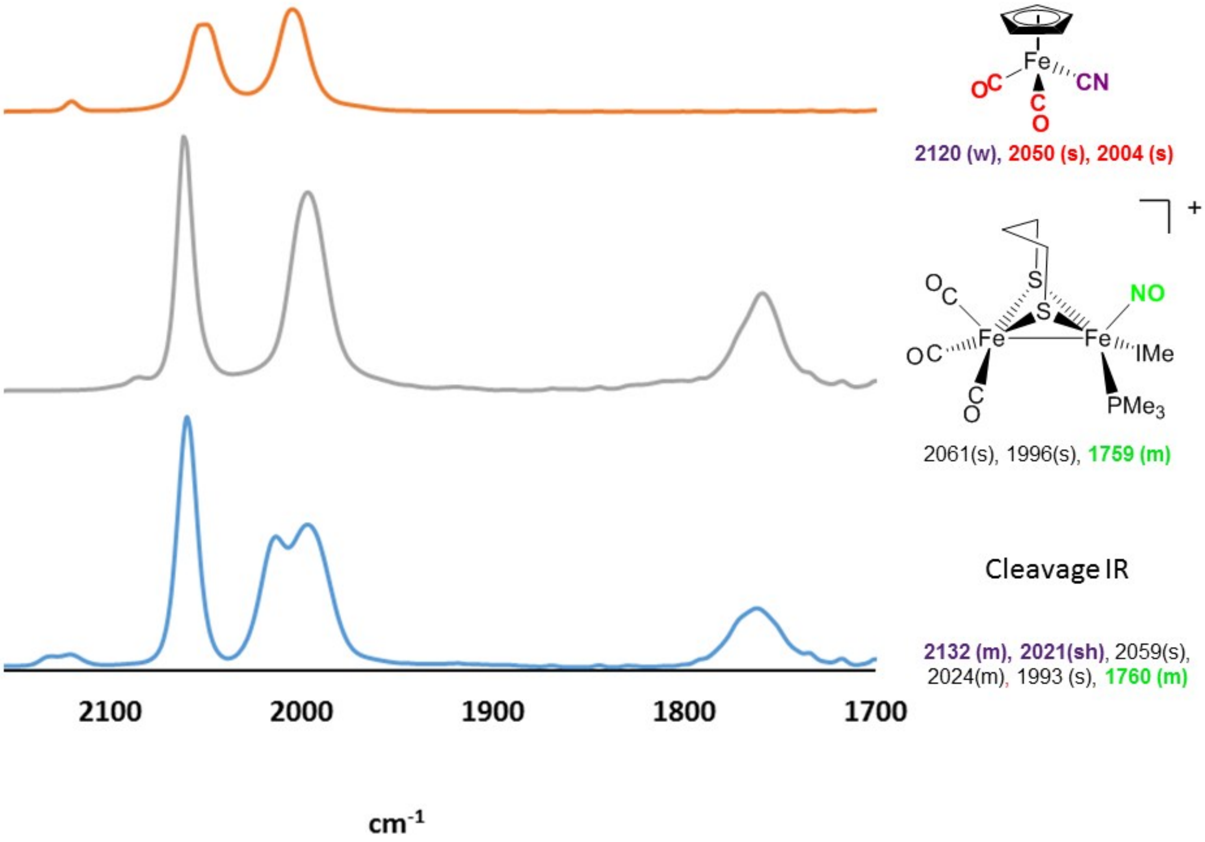


**Figure S17.** IR spectrum in THF of the cleavage of Compound **C** using $PMe_3$.

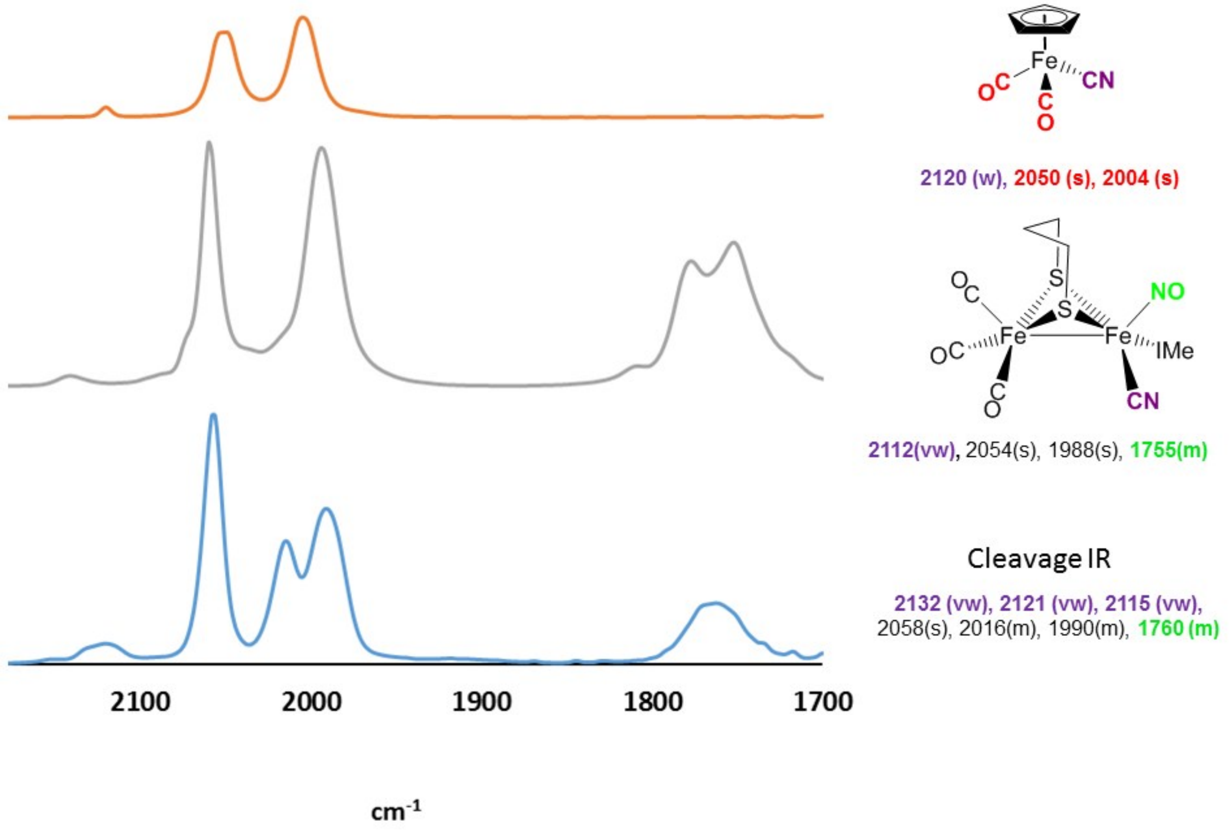


**Figure S18.** IR spectrum in THF of the cleavage of Compound **C** using tetraethylammonium cyanide.

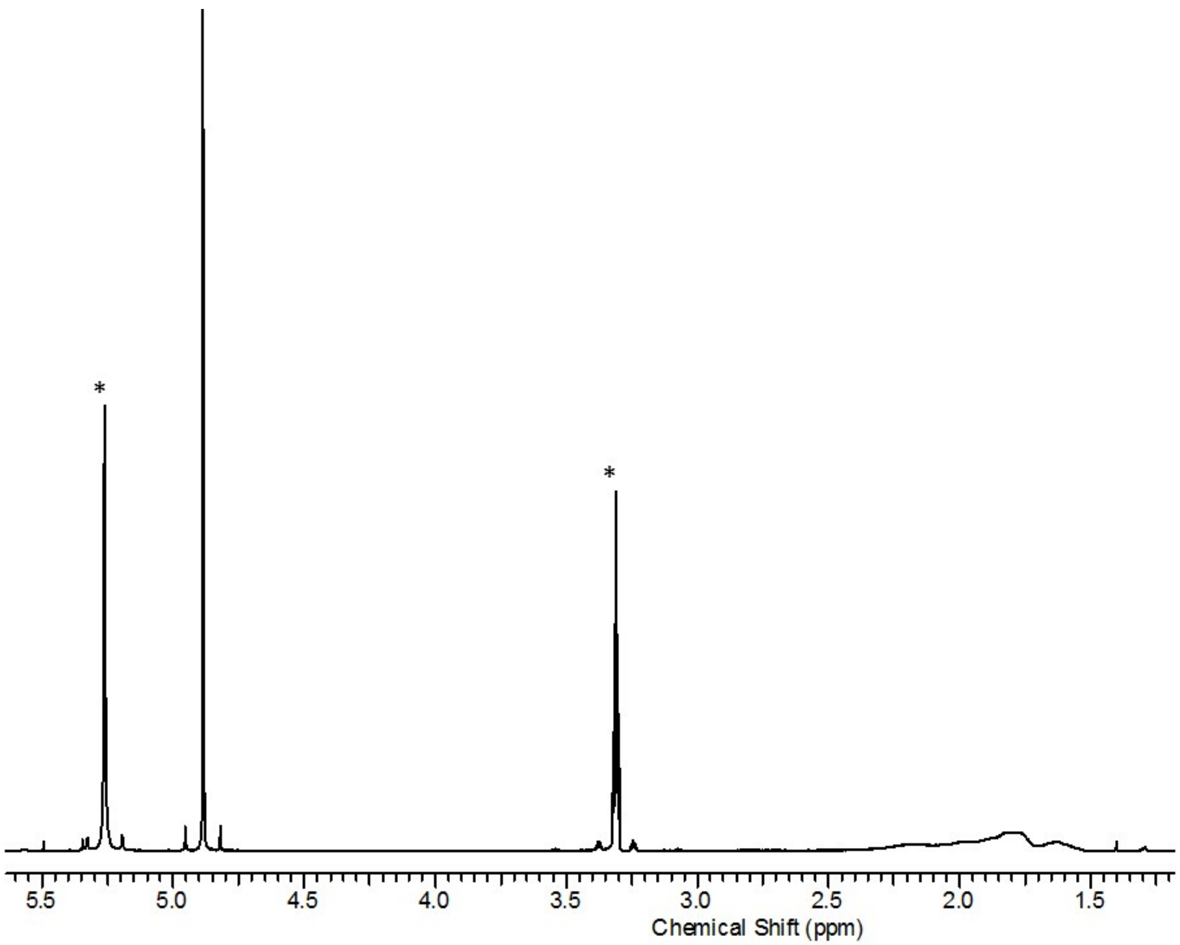


**Figure S19.** $^{1}H$ NMR of Compound **A** in $CD_3OD$. Asterisk denotes residual protonated MeOH.

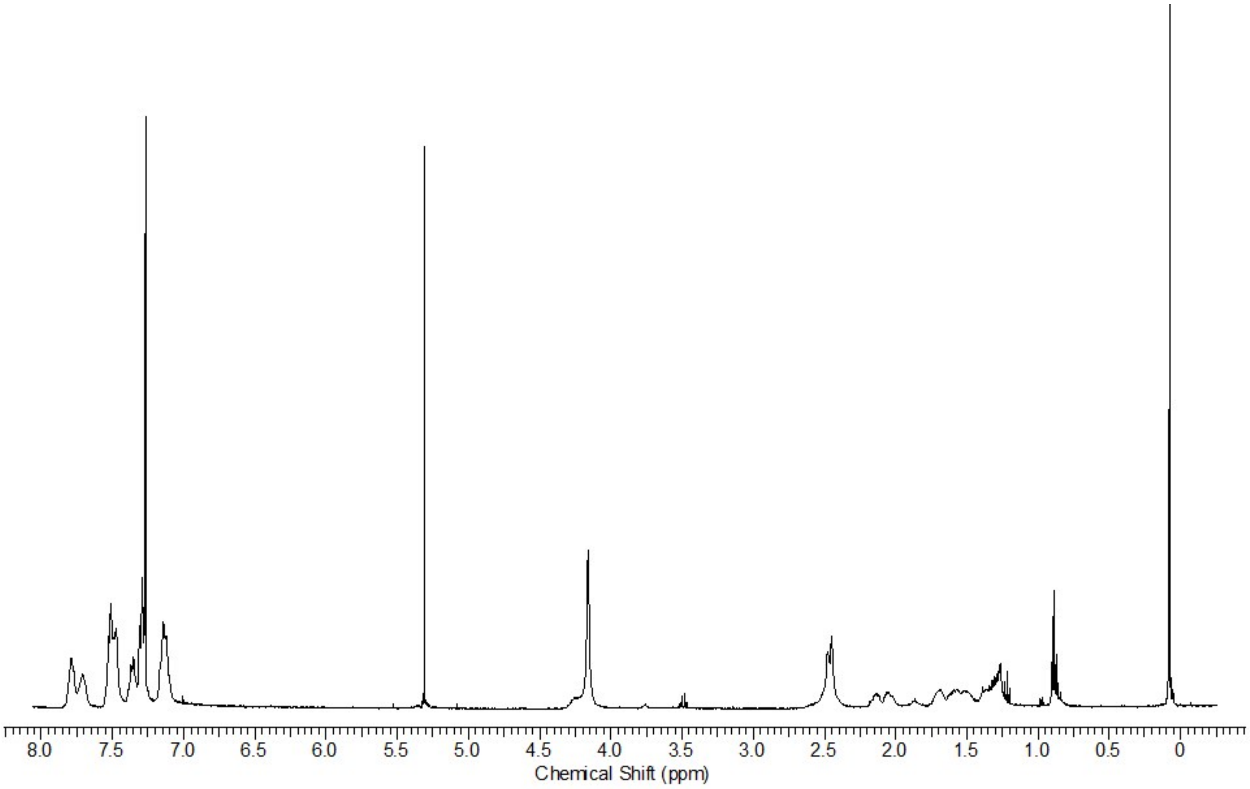


**Figure S20.** $^{1}H$ NMR of Compound **B** in $CDCl_3$.

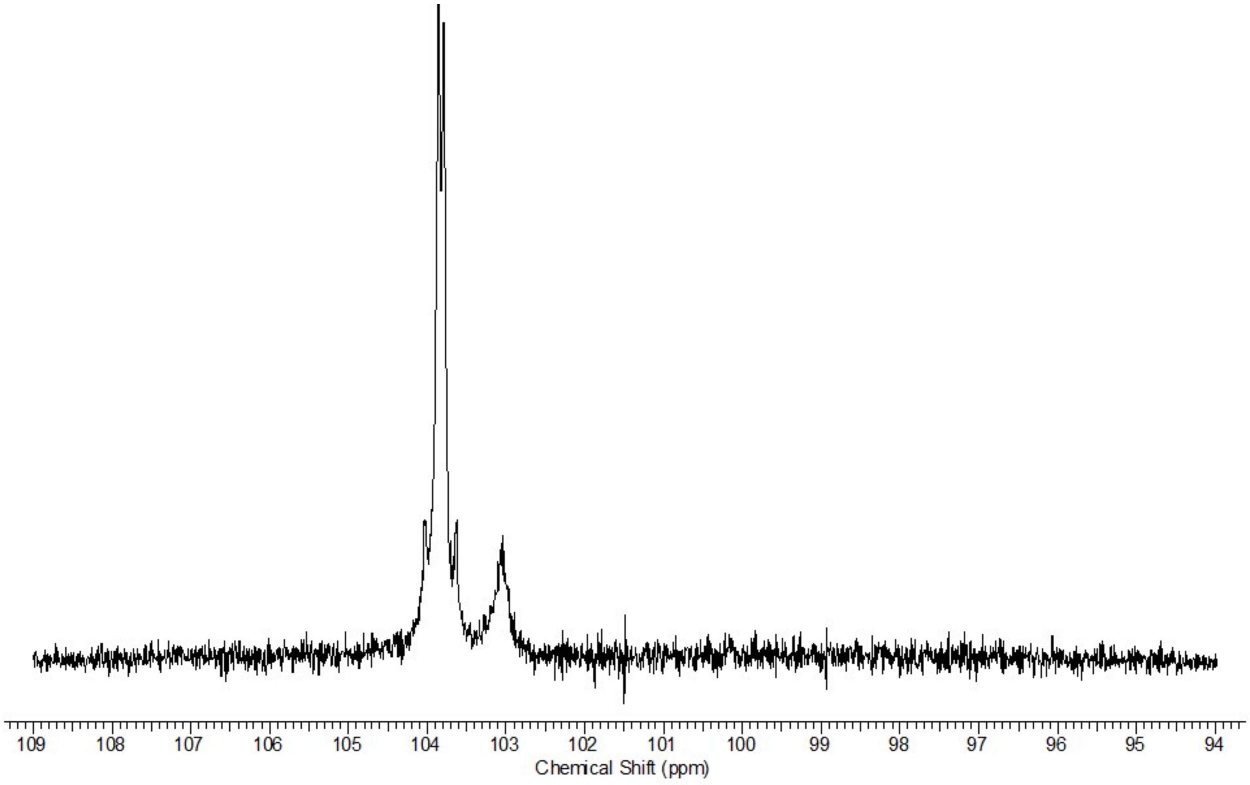


**Figure S21**. $^{31}P$ NMR of Compound **B** in $CDCl_3$.

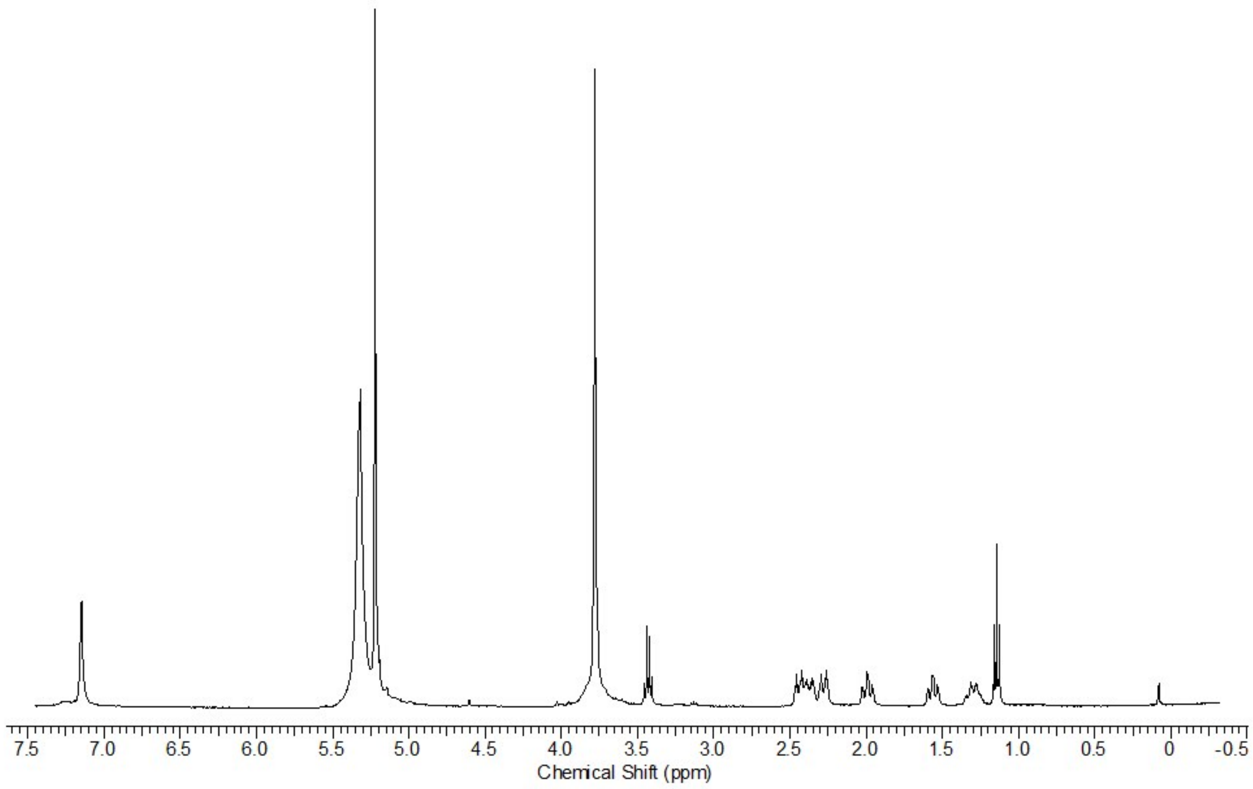


**Figure S22.** $^1$H NMR of Compound **C** in $CD_2Cl_2$.

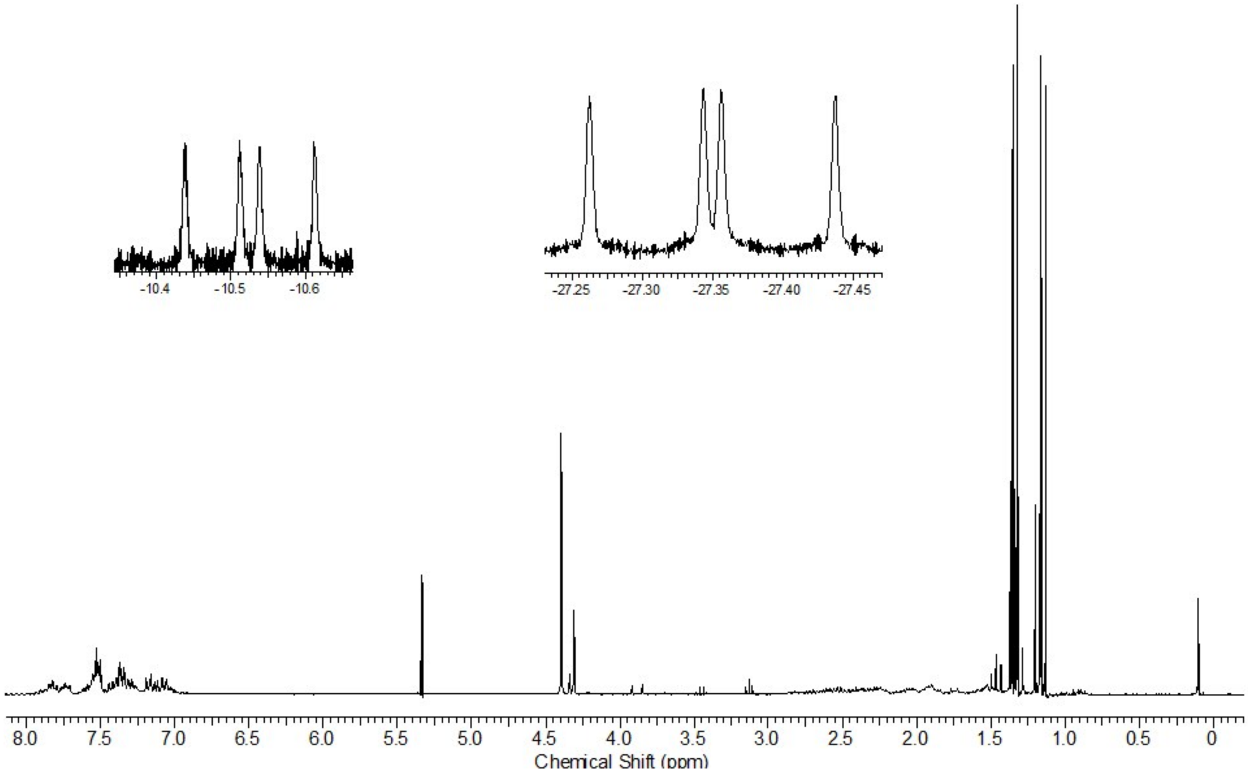


**Figure S23.** $^{1}H$ NMR of Compound **D** in $CD_2Cl_2$. The hydric upfield region is displayed in the insets.

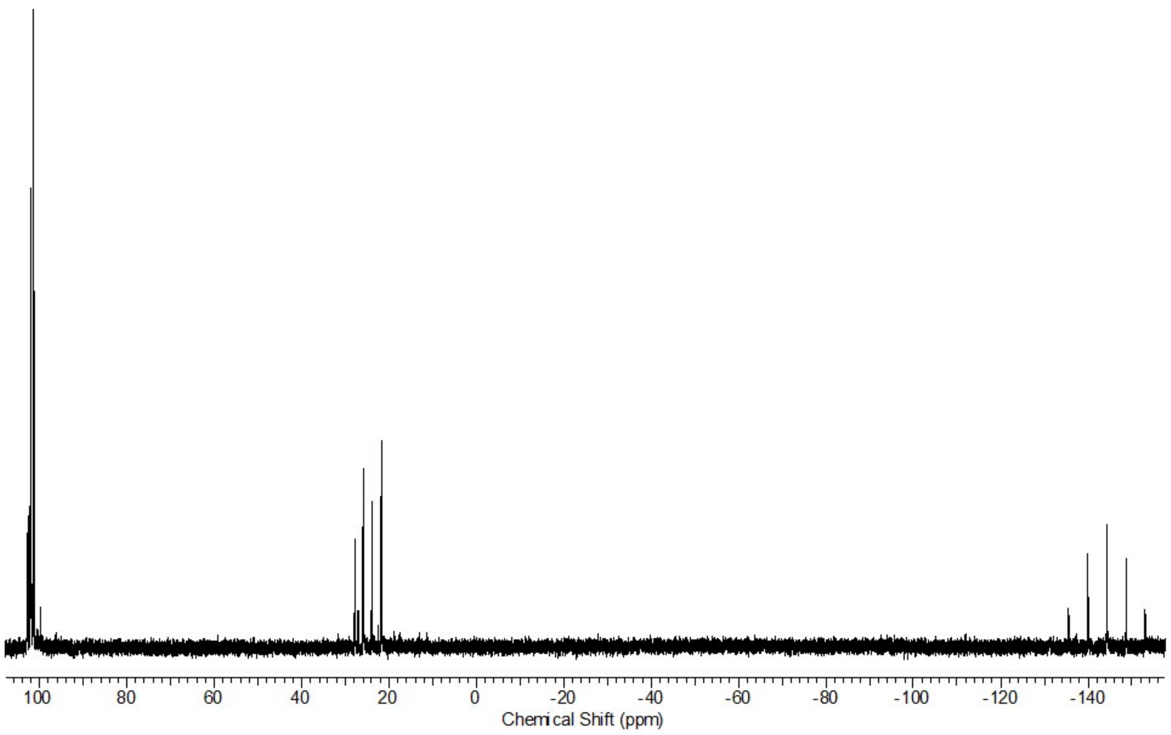


**Figure S24.** $^{31}P$ NMR of Compound **D** in $CD_2Cl_2$

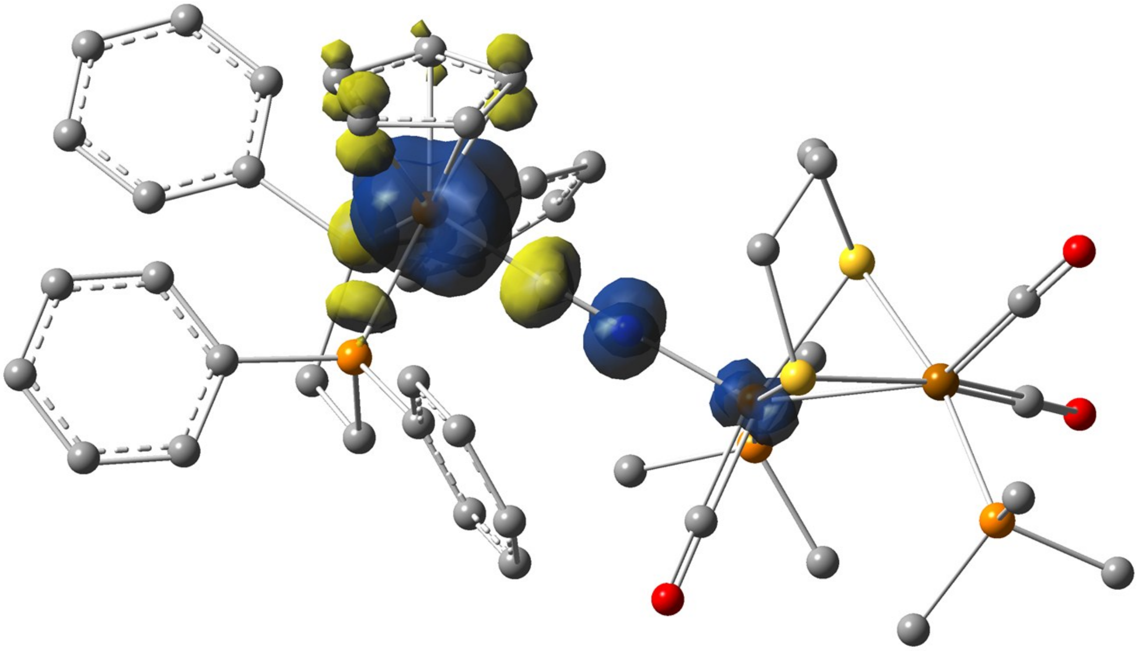

**Figure S25.** The spin density contour plot of oxidized **D**. (isovalue: 0.0025), calculated by B3LYP. The calculation s show the majority of the unpaired spin is on the iron of the mono-iron moiety, with spin density of 1.163 e, while minor excess spins are on two other irons, bridging cyanide and cyclopentadienyl. The *g* vector was derived from ca lculated *g* tensor and estimated to be (2.018, 2.068 and 2.235). Also see table S3.

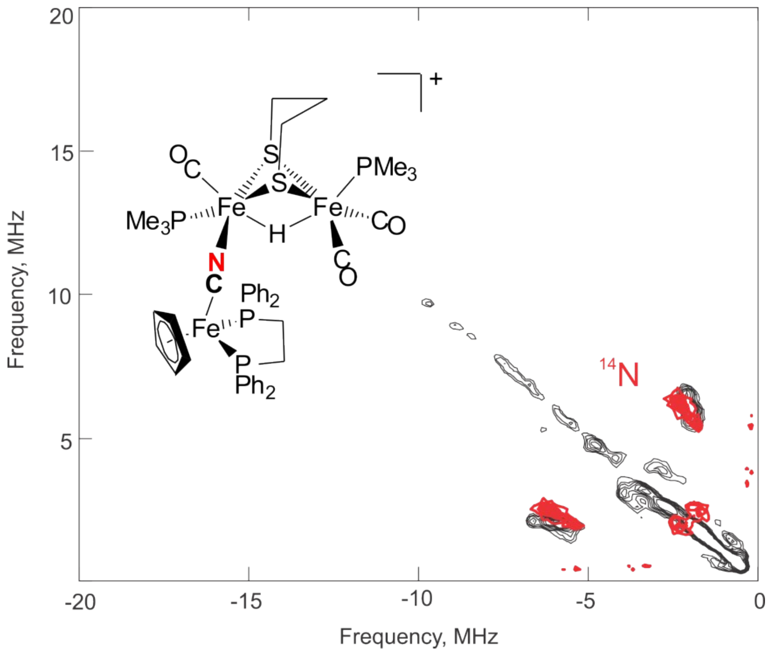


**Figure S26.** X-band HYSCORE spectrum of electrochemically oxidized (with 0.65 V) complex, $\mathbf{D}^{+}$, in DCM at 10K. Simulation (in red) parameters (with Easyspin): g (1, 2, 3) = (2.191, 2.089, 2.024); $^{14}$N HFC, $A_{iso}$ = 3 MHz, *K* = 0.5; eta = 0.1; tau = 200ns: Field position = 343.8 mT (corresponding to $g_2$); Micro wave frequency = 9.78651 GHz.

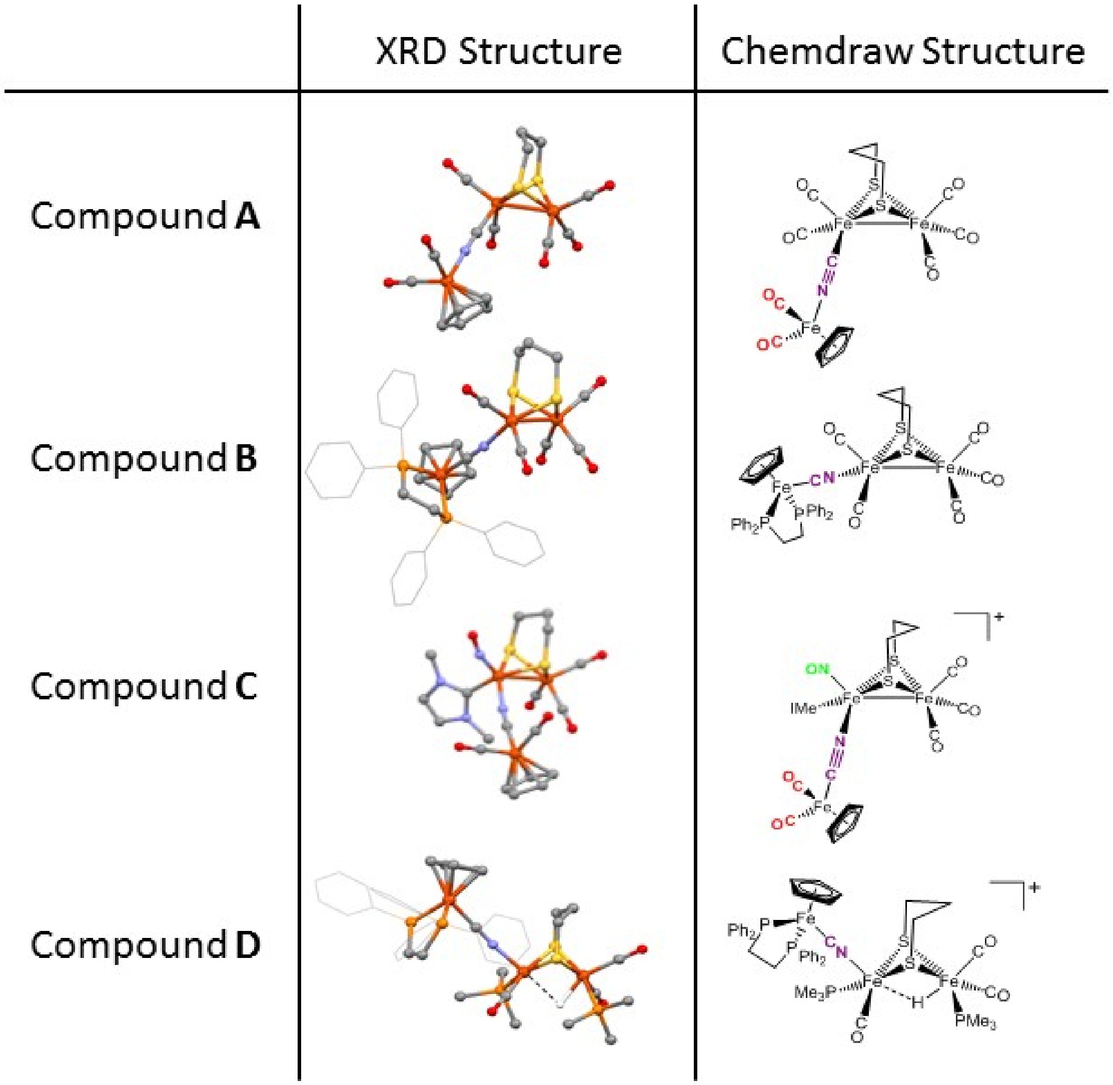


**Figure S27**. Structures for compounds **A-D** as determined by X-ray diffraction experiments with the analogous chemdraw structures. Hydrogen atoms and counter ions have been omitted for clarity and phenyl rings are in wireframe. Thermal ellipsoid plots are at 50% probability.

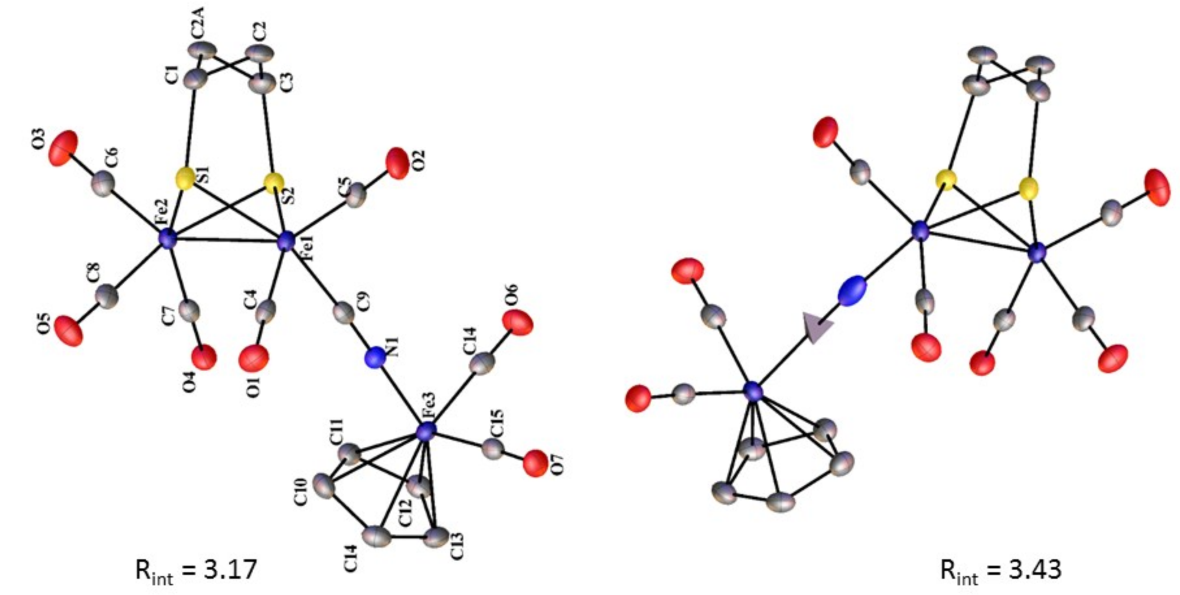


**Figure S28.** Comparison of the shape of the thermal ellipsoid plots and resulting refinement factor for the two cyanide orientations for Compound **A.** Hydrogens omitted for clarity.

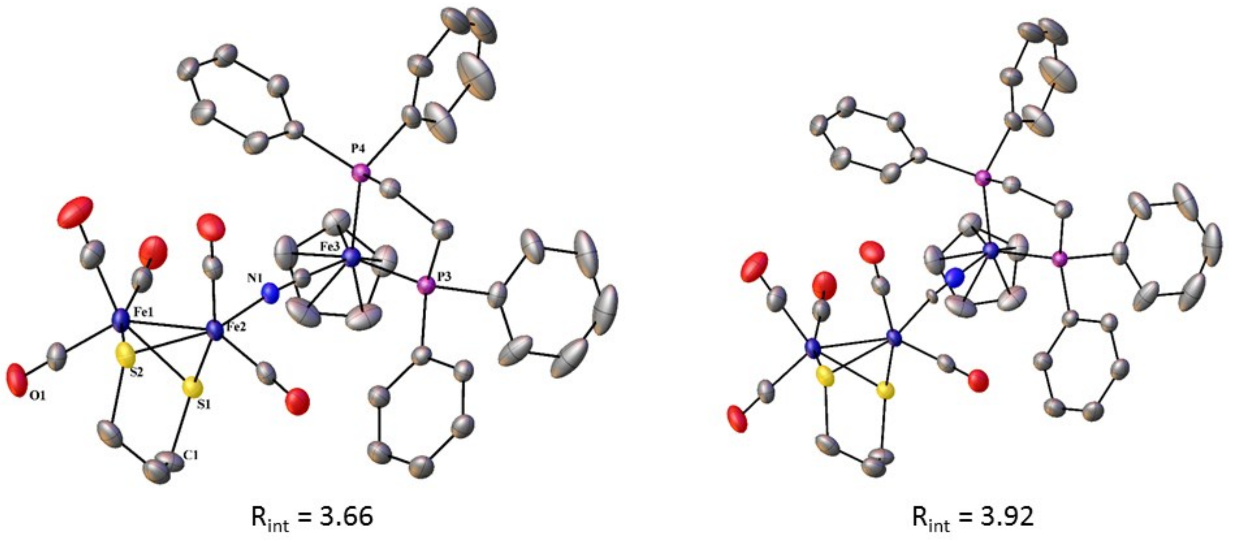


**Figure S29.** Comparison of the shape of the thermal ellipsoid plots and resulting refinement factor for the two cyanide orientations for Compound **B.** Hydrogens omitted for clarity.

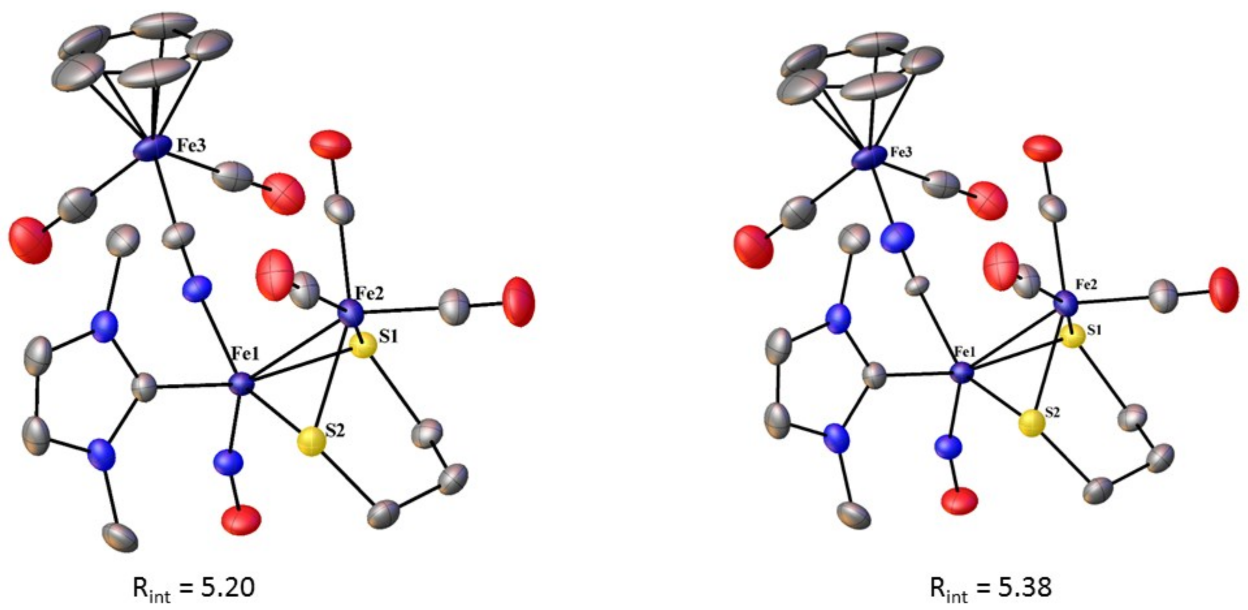


**Figure S30.** Comparison of the shape of the thermal ellipsoid plots and resulting refinement factor for the two cyanide orientations for Compound **C**. Counter ions have been omitted for clarity. Hydrogens omitted for clarity.

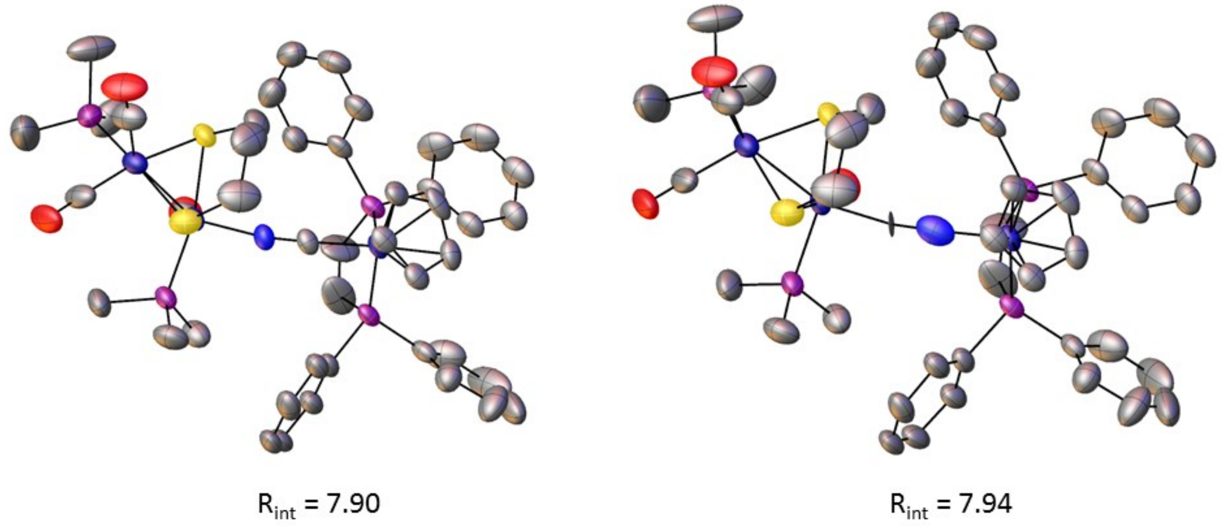


**Figure S31.** Comparison of the shape of the thermal ellipsoid plots and resulting refinement factor for the two cyanide orientations for Compound **D**. Counter ions have been omitted for clarity. Hydrogens omitted for clarity.

**Table S1.** Selected metric data of optimized structures in comparison to experimental values.

| Metric data | **Complex A** (Fe-NC-FeFe) | | | **Complex B** (Fe-CN-FeFe) | | | **Complex C** (Fe-CN-FeFe) | | | **Complex D** (Fe-CN-FeFe) | | |
|---|---|---|---|---|---|---|---|---|---|---|---|---|
| | Exp. | **Fe-NC-FeFe** | Fe-CN-FeFe | Exp. | Fe-NC-FeFe | **Fe-CN-FeFe** | Exp. | Fe-NC-FeFe | **Fe-CN-FeFe** | Exp | Fe-NC-FeFe | **Fe-CN-FeFe** |
| Fe-N | 1.930 | 1.911 | 1.928 | 1.956 | 1.921 | 1.946 | 1.972 | 1.919 | 1.949 | 1.939 | 1.936 | 1.942 |
| N-C | 1.152 | 1.177 | 1.170 | 1.158 | 1.179 | 1.179 | 1.147 | 1.174 | 1.172 | 1.172 | 1.182 | 1.184 |
| C-Fe | 1.917 | 1.881 | 1.897 | 1.876 | 1.908 | 1.878 | 1.900 | 1.917 | 1.892 | 1.887 | 1.906 | 1.889 |
| Fe-N-C | 177.6 | 176.2 | 177.5 | 170.6 | 174.8 | 175.4 | 171.4 | 175.3 | 169.6 | 171.6 | 176.0 | 173.7 |
| N-C-Fe | 177.2 | 177.7 | 177.5 | 177.1 | 177.5 | 177.0 | 177.1 | 172.9 | 177.9 | 177.4 | 175.1 | 176.0 |

Comments: Though C and N are similar in electron count and are expected to yield comparable diffractions when examined by X-ray crystallography, the metric data should be reliable regardless of the assignment of C or N. By comparing the differences between FeFe-C/N and Fe-C/N bond lengths, the experimental assignments of complexes **B**-**D** are confirmed by calculations. For complex **A**, the difference is too small to make a judgment.

**Table S2.** Computational IR frequencies in comparison with experimental values

| Complex | Orientation | Vibrational frequency ($cm^{-1}$) | | |
|---|---|---|---|---|
| | | CN | NO | CO (Those from monoiron unit are underlined) |
| A | exp[a] | 2128 | - | 1930, 1955,1986, 2026, 2039, 2069 |
| | **Fe-NC-FeFe** | 2130 | - | 1947, 1948, 1966, 1986, 2018, 2023, 2058 |
| | Fe-CN-FeFe | 2173 | - | 1944, 1945, 1963, 1986, 2017, 2024, 2055 |
| B | exp | 2120 | - | 1919, 1947, 1980, 2028, 2035 |
| | Fe-NC-FeFe | 2102 | - | 1932, 1954, 1961, 1976, 2021 |
| | **Fe-CN-FeFe** | 2100 | - | 1929, 1952, 1957, 1975, 2020 |
| C | exp[a] | 2154 | 1755 | 1995, 2024, 2060, 2067 |
| | Fe-NC-FeFe | 2146 | 1810 | 1988, 2002, 2023, 2051, 2063 |
| | **Fe-CN-FeFe** | 2155 | 1795 | 1986, 2002, 2023, 2051, 2060 |
| D | exp | 2081 | - | 1949, 1964, 2020 |
| | Fe-NC-FeFe | 2078 | - | 1941, 1972, 2016 |
| | **Fe-CN-FeFe** | 2063 | - | 1939, 1970, 2014 |

a. The origins of CO frequencies cannot be distinguished solely based on frequencies.

Generally the calculated frequencies match the experimental values with an error less than 20 $cm^{-1}$. The differences between two linkage isomers are trivial therefore the determination of the structure by IR frequencies is feasible.

**Table S3.** Experimental and computational EPR parameters for complex $D^+$.

| Orientation | Hyperfine constants $A_{xyz}$ (MHz) | | | | Quadrupole Con-stant *K* (MHz) |
|---|---|---|---|---|---|
| | Two $^{31}P$ (*a* = 100 %) on the mono-iron unit | | $^{13}C$ (*a* = 1.1 %) | $^{14}N$ (*a* = 99.6 %) | $^{14}N$ (*I* = 1.0) |
| CW EPR (Exp.) | - 97, -60, -97 | -90, -50, -90 | Not detected | --- | -- |
| HYSCORE (Exp.)* | -- | -- | -- | 2, 3, 4 | 0.5 |
| Fe-NC-FeFe (Calc.) | -76.02, -77.58, -110.43 | -76.55, -77.15, -110.67 | -0.70, -3.68, 9.88 | -8.63, -9.67, -10.19 | -0.34 |
| **Fe-CN-FeFe** (Calc.) | -85.86, -87.37, -122.74 | -84.75, -85.72, -120.85 | -43.85, -52.11, -59.49 | -1.55, -2.27, 7.94 | -0.69 |

The hyperfine coupling of $^{14}N$ in the Fe-CN-FeFe isomer is calculated to be relatively small as expected because of the distance between N and the spin center (mono-iron unit). The hyperfine coupling of $^{13}C$ is hard to observe because of the low natural abundance of $^{13}C$. The hyperfine coupling constants of $^{31}P$ match fitted values from simulations to the same extent, regardless of the orientation of CN.

*Simulation parameters are as shown presenting the range of the HFC components. Field dependent HYSCORE is required in order to obtain quantitative anisotropic components of the $^{14}N$ HFC which was not possible due to sensitivity issues at $g_1$ and $g_3$.

**Table S4.** Energy profiles (Gibbs free energy in kcal/mol) of cyanide flipping with H-bond providers

| H-bond provider (P) | Fe-CN-FeFe | Fe-CN-FeFe + P | TS( + P) | Fe-NC-FeFe + P | Fe-NC-FeFe |
|---|---|---|---|---|---|
| None [a] | 0 | -- | 34.1 | -- | 1.0 |
| $H_2O$ | 0 | 6.0 | 34.3 | 7.5 | 1.0 |
| $(H_2O)_2$ | 0 | 9.8 | 43.2 | 12.1 | 1.0 |
| Urea | 0 | 4.2 | 39.4 | 6.0 | 1.0 |
| $Hpy^+$ | 0 | 2.8 | 36.1 | 4.8 | 1.0 |

a. In addition to thermal and solvation corrections, empirical dispersion (GD3BJ) is also added to fully evaluate the contributions from possible H-bonds. Therefore the values of the CN-bridged complexes are slightly different from those in the text without the dispersion.

**Table S5.** Crystal data and structure refinement for Compound **A**

| | | |
|---|---|---|
| Identification code | cn | |
| Empirical formula | C16 H11 Fe3 N O7 S2 | |
| Formula weight | 560.93 | |
| Temperature | 110.15 K | |
| Wavelength | 0.71073 Å | |
| Crystal system | Triclinic | |
| Space group | P -1 | |
| Unit cell dimensions | a = 7.7263(14) Å | $\alpha$= 94.504(10)°. |
| | b = 11.203(2) Å | $\beta$= 108.019(10)°. |
| | c = 11.900(2) Å | $\gamma$ = 93.687(10)°. |
| Volume | 972.3(3) $Å^3$ | |
| Z | 2 | |
| Density (calculated) | 1.916 $Mg/m^3$ | |
| Absorption coefficient | 2.463 $mm^{-1}$ | |
| F(000) | 560 | |
| Crystal size | 0.12 x 0.1 x 0.08 $mm^3$ | |
| Theta range for data collection | 1.809 to 27.575°. | |
| Index ranges | -10<=h<=9, -14<=k<=14, 0<=l<=15 | |
| Reflections collected | 4458 | |
| Independent reflections | 4458 [R(int) = ?] | |
| Completeness to theta = 25.242° | 99.7 % | |
| Absorption correction | Semi-empirical from equivalents | |
| Max. and min. transmission | 0.745 and 0.581 | |
| Refinement method | Full-matrix least-squares on $F^2$ | |
| Data / restraints / parameters | 4458 / 6 / 267 | |
| Goodness-of-fit on $F^2$ | 1.051 | |
| Final R indices [I>2sigma(I)] | R1 = 0.0317, wR2 = 0.0756 | |
| R indices (all data) | R1 = 0.0380, wR2 = 0.0788 | |
| Extinction coefficient | n/a | |
| Largest diff. peak and hole | 0.516 and -0.467 e.$Å^{-3}$ | |

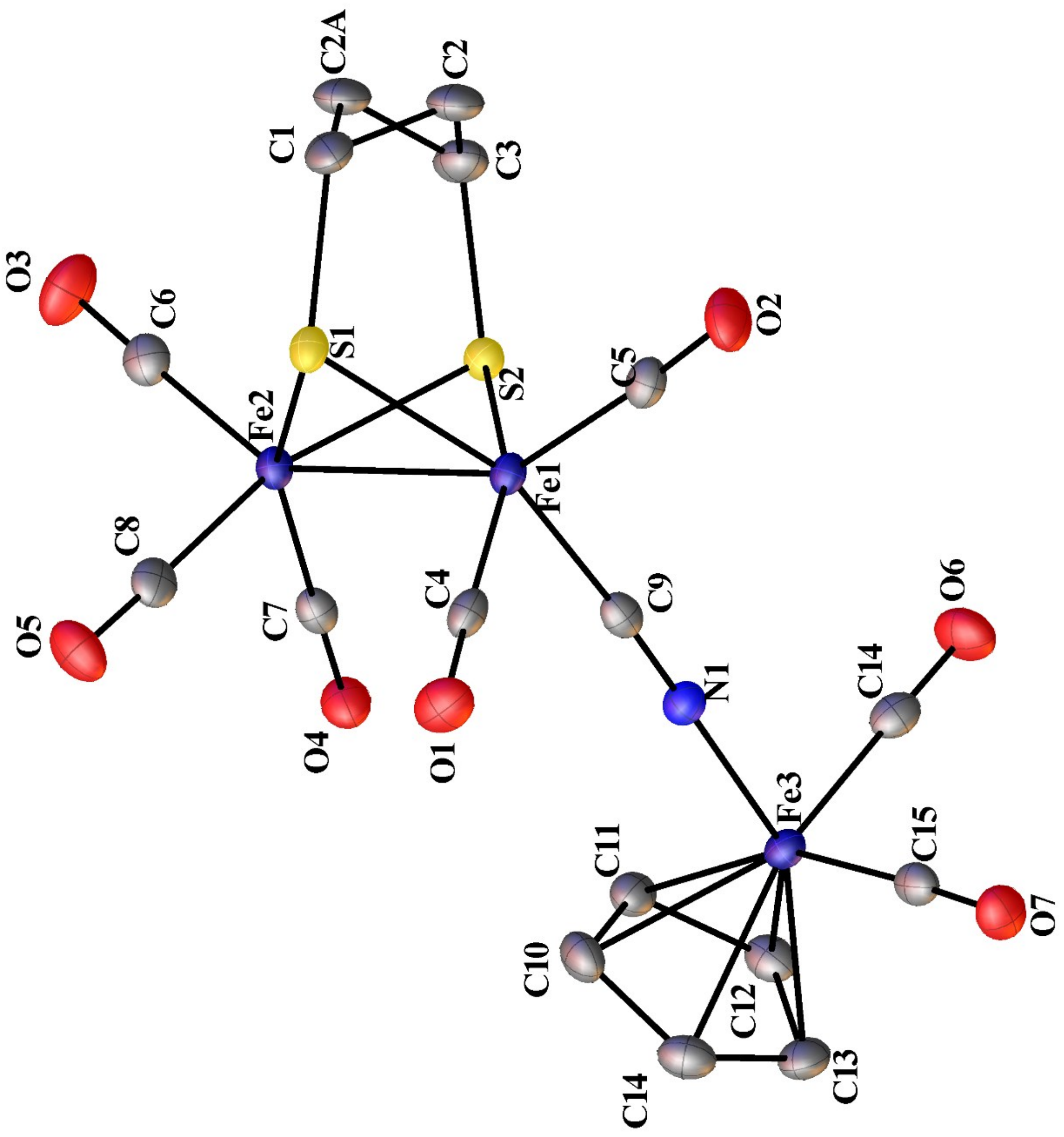


**Figure S32.** Thermal ellipsoid plot at 50% probability for Compound **A**. Hydrogens omitted for clarity.

**Table S6**. Crystal data and structure refinement for Compound **B**.

| | | |
|---|---|---|
| Identification code | pdtisomer | |
| Empirical formula | C40 H35 Fe3 N O5 P2 S2 | |
| Formula weight | 903.30 | |
| Temperature | 150.15 K | |
| Wavelength | 0.71073 Å | |
| Crystal system | Triclinic | |
| Space group | P-1 | |
| Unit cell dimensions | a = 11.3103(15) Å | $\alpha$= 105.449(5)°. |
| | b = 12.9416(18) Å | $\beta$= 100.216(5)°. |
| | c = 15.479(2) Å | $\gamma$ = 110.921(4)°. |
| Volume | 1944.2(5) Å$^3$ | |
| Z | 2 | |
| Density (calculated) | 1.543 Mg/m$^3$ | |
| Absorption coefficient | 1.339 mm$^{-1}$ | |
| F(000) | 924 | |
| Crystal size | 0.15 x 0.14 x 0.1 mm$^3$ | |
| Theta range for data collection | 2.023 to 27.898°. | |
| Index ranges | -14<=h<=14, -16<=k<=16, -20<=l<=20 | |
| Reflections collected | 87852 | |
| Independent reflections | 9112 [R(int) = 0.0396] | |
| Completeness to theta = 25.242° | 99.9 % | |
| Absorption correction | Semi-empirical from equivalents | |
| Max. and min. transmission | 0.7456 and 0.6533 | |
| Refinement method | Full-matrix least-squares on F$^2$ | |
| Data / restraints / parameters | 9112 / 90 / 478 | |
| Goodness-of-fit on F$^2$ | 1.037 | |
| Final R indices [I>2sigma(I)] | R1 = 0.0366, wR2 = 0.0864 | |
| R indices (all data) | R1 = 0.0469, wR2 = 0.0946 | |
| Extinction coefficient | n/a | |
| Largest diff. peak and hole | 1.370 and -0.472 e.Å$^{-3}$ | |

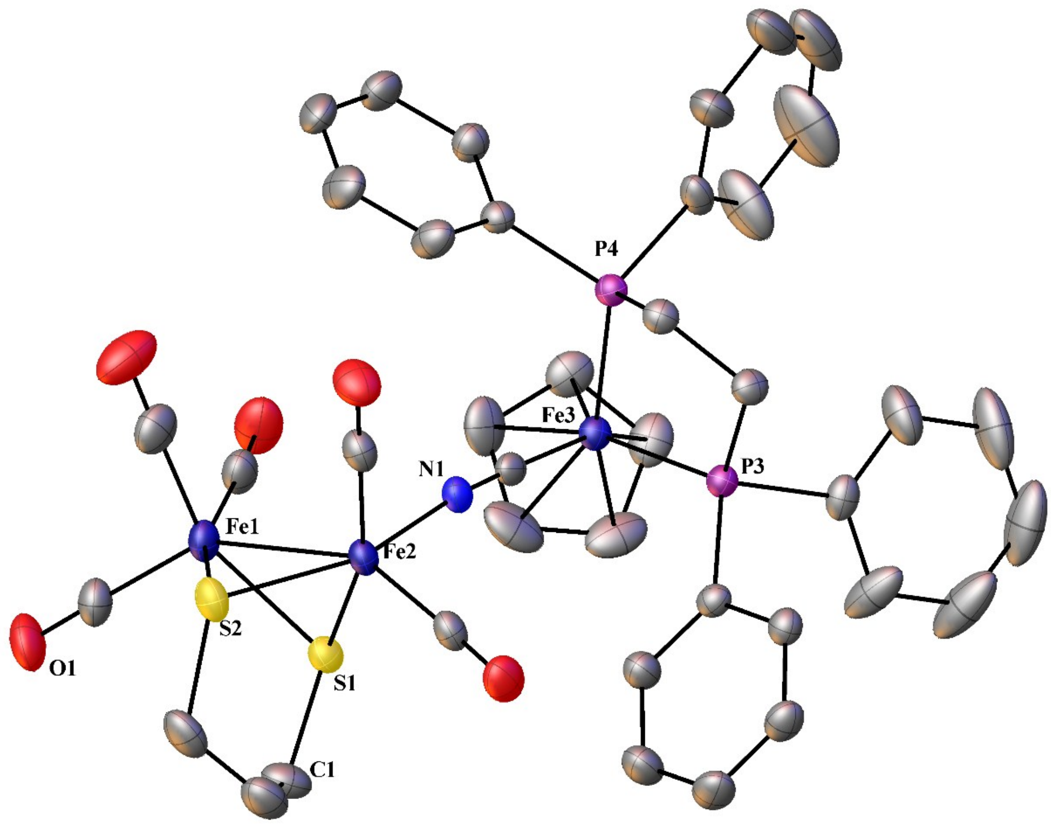


**Figure S33.** Thermal ellipsoid plot at 50% probability for **B**. Hydrogens omitted for clarity.

**Table S7.** Crystal data and structure refinement for Compound **C**.

| | | |
|---|---|---|
| Identification code | 8314 | |
| Empirical formula | C20.20 H22.23 B F4 Fe3 N4.60 O6.72 S2 | |
| Formula weight | 755.40 | |
| Temperature | 110.15 K | |
| Wavelength | 0.71073 Å | |
| Crystal system | Triclinic | |
| Space group | P-1 | |
| Unit cell dimensions | a = 10.4593(5) Å | $\alpha$= 76.988(2)°. |
| | b = 11.6085(6) Å | $\beta$= 85.341(2)°. |
| | c = 12.2560(6) Å | $\gamma$ = 81.750(2)°. |
| Volume | 1433.02(12) $Å^3$ | |
| Z | 2 | |
| Density (calculated) | 1.751 $Mg/m^3$ | |
| Absorption coefficient | 1.717 $mm^{-1}$ | |
| F(000) | 761 | |
| Crystal size | 0.142 x 0.10 x 0.038 $mm^3$ | |
| Theta range for data collection | 1.970 to 30.797°. | |
| Index ranges | -14<=h<=14, -16<=k<=16, -17<=l<=17 | |
| Reflections collected | 46532 | |
| Independent reflections | 8411 [R(int) = 0.0436] | |
| Completeness to theta = 25.242° | 99.8 % | |
| Absorption correction | Semi-empirical from equivalents | |
| Max. and min. transmission | 0.7461 and 0.6145 | |
| Refinement method | Full-matrix least-squares on $F^2$ | |
| Data / restraints / parameters | 8411 / 66 / 369 | |
| Goodness-of-fit on $F^2$ | 1.055 | |
| Final R indices [I>2sigma(I)] | R1 = 0.0520, wR2 = 0.1487 | |
| R indices (all data) | R1 = 0.0658, wR2 = 0.1629 | |
| Extinction coefficient | n/a | |
| Largest diff. peak and hole | 2.007 and -1.856 e.$Å^{-3}$ | |

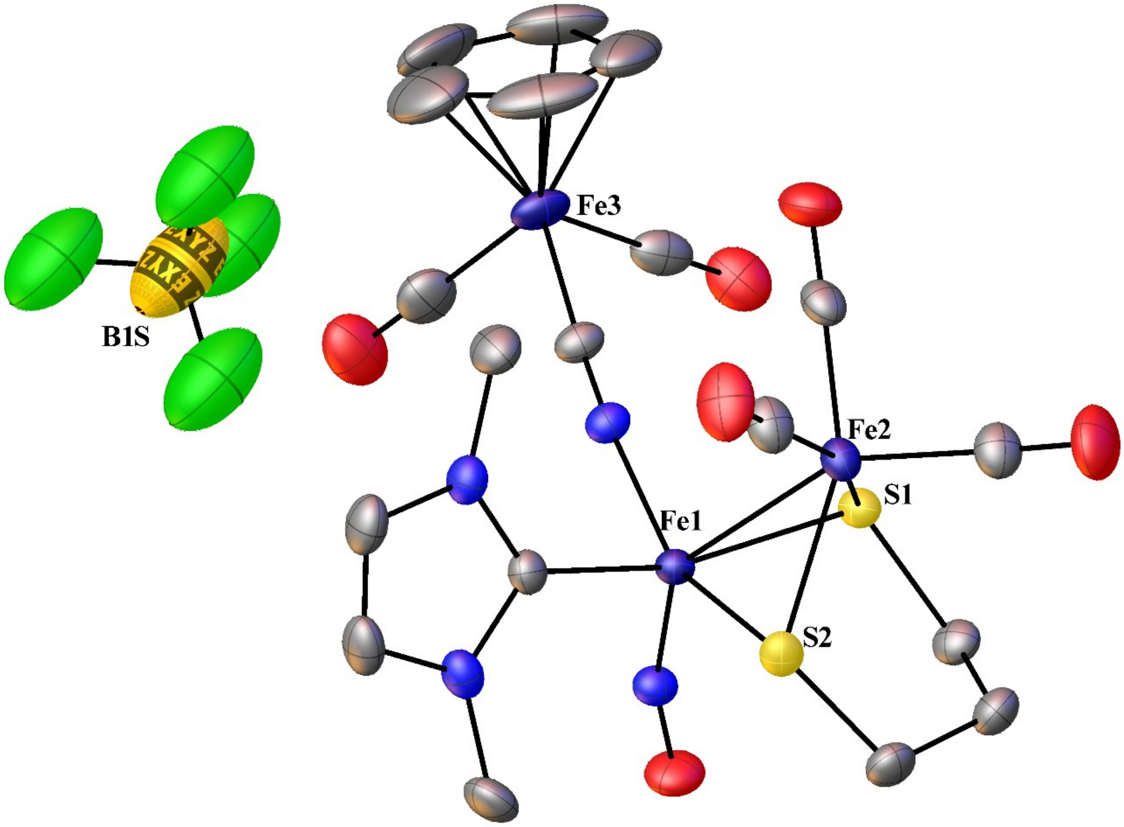


**Figure S34.** Thermal ellipsoid plot at 50% probability for **C**. Hydrogens omitted for clarity.

**Table S8.** Crystal data and structure refinement for Compound **D**.

| | | |
|---|---|---|
| Identification code | twin4 | |
| Empirical formula | C80 H75 B F24 Fe3 N O4 P4 S2 | |
| Formula weight | 1936.77 | |
| Temperature | 150.15 K | |
| Wavelength | 0.71073 Å | |
| Crystal system | Triclinic | |
| Space group | P-1 | |
| Unit cell dimensions | a = 13.352(5) Å | $\alpha$= 92.828(5)°. |
| | b = 13.623(5) Å | $\beta$= 94.410(5)°. |
| | c = 24.074(8) Å | $\gamma$ = 99.584(5)°. |
| Volume | 4296(3) $Å^3$ | |
| Z | 2 | |
| Density (calculated) | 1.497 $Mg/m^3$ | |
| Absorption coefficient | 0.723 $mm^{-1}$ | |
| F(000) | 1970 | |
| Crystal size | 0.15 x 0.09 x 0.08 $mm^3$ | |
| Theta range for data collection | 2.138 to 23.850°. | |
| Index ranges | ?<=h<=?, ?<=k<=?, ?<=l<=? | |
| Reflections collected | ? | |
| Independent reflections | 13129 [R(int) = ?] | |
| Completeness to theta = 25.242° | 84.5 % | |
| Absorption correction | Semi-empirical from equivalents | |
| Max. and min. transmission | 0.744 and 0.622 | |
| Refinement method | Full-matrix least-squares on $F^2$ | |
| Data / restraints / parameters | 13129 / 0 / 1080 | |
| Goodness-of-fit on $F^2$ | 1.082 | |
| Final R indices [I>2sigma(I)] | R1 = 0.0790, wR2 = 0.2135 | |
| R indices (all data) | R1 = 0.1091, wR2 = 0.2317 | |
| Extinction coefficient | n/a | |
| Largest diff. peak and hole | 2.070 and -0.534 e.$Å^{-3}$ | |

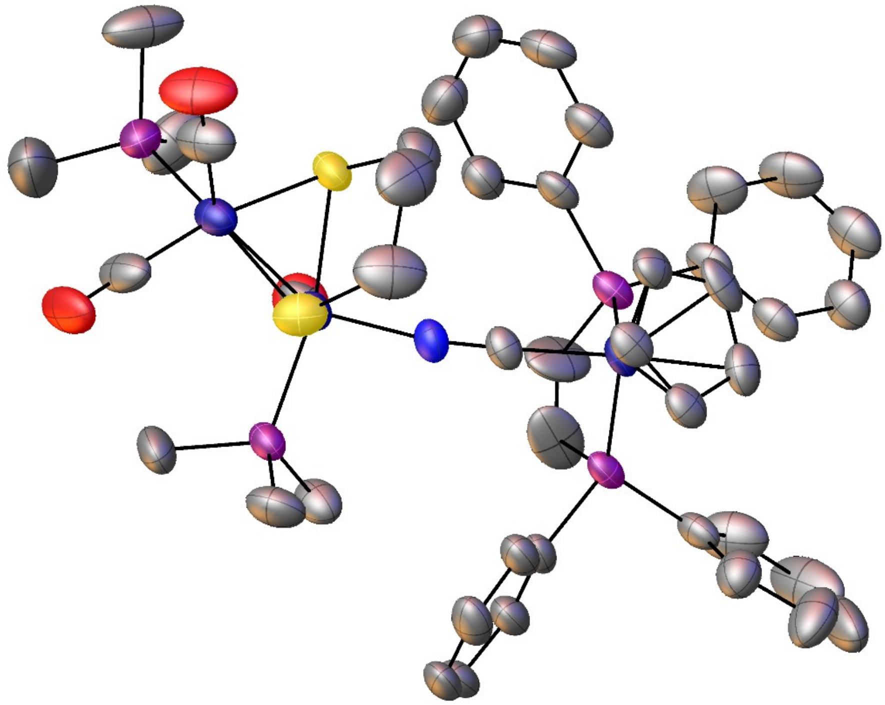

**Figure S35.** Thermal ellipsoid plot at 50% probability for **D**. Hydrogens and counter ion omitted for clarity.
